# The Effects of Agent Type and Feedback Style on Self-Directed Learning: A Mixed-Methods Study

**DOI:** 10.3390/bs16071069

**Published:** 2026-06-30

**Authors:** Xue Han, Jing Cao, Zi Wang, Heng Luo

**Affiliations:** Faculty of Artificial Intelligence in Education, Central China Normal University, Wuhan 430079, China; hanx@mails.ccnu.edu.cn (X.H.); caojing@mails.ccnu.edu.cn (J.C.); 18836222992@mails.ccnu.edu.cn (Z.W.)

**Keywords:** artificial intelligence, agent type, feedback style, self-directed learning, self-regulation, human–AI interaction, mixed methods

## Abstract

This study examines how agent type (customized vs. general-purpose) and feedback style (Socratic vs. directive) are associated with learners’ engagement with artificial intelligence (AI)-generated feedback in self-directed learning (SDL), with particular attention to patterns in feedback quality, self-regulatory behaviors, learning experiences, and learning outcomes. A 2 × 2 mixed factorial experiment was conducted with 51 postgraduate students who completed two instructional design tasks under different feedback conditions. Quantitative results indicated that customized agents generated feedback with higher accuracy and specificity than general-purpose agents. Socratic feedback was associated with stronger comprehension monitoring, whereas directive feedback was associated with higher cognitive load. A significant interaction suggested that the advantage of customized agents in learning outcomes, operationalized as short-term task improvement, emerged under directive feedback but not under Socratic feedback. Qualitative analysis indicated that Socratic prompts encouraged deeper, logic-oriented reflection, whereas directive feedback provided actionable guidance that facilitated task completion. Learners adopted feedback selectively based on perceived accuracy, and trust in customized agents was higher when feedback was clear and contextually aligned. These findings suggest that the effectiveness of AI-generated feedback is shaped not only by agent type and feedback style but also by how learners evaluate and use feedback.

## 1. Introduction

In higher education and online learning environments, self-directed learning (SDL) has become increasingly important (Sun et al., 2023). Because SDL requires learners to set goals, monitor progress, and adjust strategies on their own, feedback plays a central role in supporting learning. High-quality feedback helps learners identify gaps, revise strategies, and sustain task progress (Hattie & Timperley, 2007; Nicol & Macfarlane-Dick, 2006). However, providing effective feedback in SDL remains challenging. Teacher feedback is often constrained by time, scale, and task complexity, making timely, specific, and sustained support difficult (Brandmo & Gamlem, 2025). In addition, feedback is effective only when learners can understand, evaluate, and use it, which depends on their feedback literacy and self-regulatory capacity (Carless & Boud, 2018; Panadero, 2017). Therefore, in SDL, the key issue is not simply whether feedback is available, but whether it can support learners’ ongoing learning in meaningful ways.

Recent advances in generative artificial intelligence (AI), particularly large language models (LLMs), have created new possibilities for providing scalable, personalized, and context-sensitive feedback in educational settings (Huber et al., 2024; Zhai, 2023). In this context, educational agents capable of dialogue, feedback, and task support are increasingly positioned as intermediaries between learners and knowledge (Córdova-Esparza, 2025; García-Méndez et al., 2025; Sharma et al., 2025). In self-directed learning settings, such agents can be embedded into learners’ task execution processes to provide ongoing and immediate feedback, thereby helping to address some of the limitations of traditional feedback in terms of timeliness, accessibility, and personalization.

However, the integration of educational agents does not necessarily lead to better learning processes or outcomes. Previous studies have shown that AI-generated feedback may still have limitations in reliability, accuracy, instructional depth, and contextual alignment (Jasin et al., 2023; Lin & Chang, 2023). More importantly, whether AI feedback supports or hinders SDL may depend largely on how it is designed. Although continuous and structured AI feedback can reduce task difficulty and improve efficiency, it may also partially replace learners’ own judgment and evaluative processes, thereby weakening reflective engagement and learner agency (Tian & Zhou, 2020; Zhan et al., 2025). Thus, the value of AI-supported feedback lies not only in its availability or quality, but also in whether its design sustains learners’ cognitive engagement during learning.

From this perspective, agent type and feedback style are two key design factors that may shape learners’ engagement with AI-generated feedback in self-directed learning. Agent type concerns whether feedback is provided by a customized agent with embedded domain knowledge or by a general-purpose agent that relies mainly on the base model’s broad generalization capabilities. Customized agents may provide more accurate, contextually grounded, and task-aligned feedback, thereby strengthening learners’ perceptions of expertise, credibility, and relevance (Goldshtein et al., 2025; Omirali et al., 2025), whereas general-purpose agents may offer greater flexibility and broader responsiveness across varied learning situations (Kinder et al., 2025). Feedback style concerns whether feedback is delivered through guiding questions that encourage reflection or through direct suggestions that support revision. These different styles are likely to shape how learners allocate cognitive resources and engage in metacognitive processing during task execution (Castro-Alonso et al., 2021; T. Luo & Liu, 2023). Together, agent type and feedback style may influence feedback quality, self-regulatory behaviors, learning experiences, and learning outcomes by shaping how learners interpret, trust, and use AI-generated feedback. Importantly, the effects of these two design factors may not be independent. The value of domain-grounded feedback may depend on how that feedback is delivered and on the extent to which a particular feedback style requires learners to interpret, evaluate, or directly implement the suggestions provided.

Although previous research has examined AI-supported feedback from multiple perspectives, most studies have focused on only one design factor at a time. For example, existing work has separately examined the effectiveness of general AI in providing language feedback (Wu et al., 2025), the potential of customized systems in specific disciplines (Bauer et al., 2025; Gabrovšek & Rihtaršič, 2025), and the effects of Socratic feedback on students’ academic performance and reflective thinking (Xi et al., 2026). This fragmented approach limits understanding of whether one design feature works differently when combined with another. In particular, few studies have directly examined whether the effects of customized versus general-purpose agents vary across Socratic and directive feedback conditions. Consequently, existing research provides limited evidence regarding the possible alignment between the knowledge configuration of an AI agent and the way in which its feedback is delivered. In addition, much of the existing research has emphasized outcome measures, such as improvements in learning performance, while paying less attention to how learners interpret, evaluate, and apply AI-generated feedback in authentic task contexts. Such an outcome-oriented approach may obscure the processes through which feedback supports learning, including learners’ monitoring of their understanding, judgments of feedback credibility, selective adoption or rejection of suggestions, and experiences of cognitive demand (Carless & Boud, 2018; Winstone et al., 2017). Moreover, relatively few studies have examined feedback quality, self-regulatory behaviors, learning experiences, and task improvement within the same analytical framework. Consequently, the effects of different AI feedback designs on self-directed learning remain insufficiently understood.

To address these gaps, the present study investigates how agent type and feedback style shape learners’ engagement with AI-generated feedback in self-directed learning. Specifically, a 2 × 2 mixed experimental design was adopted to examine the main and interaction effects of agent type (customized vs. general-purpose) and feedback style (Socratic vs. directive) on feedback quality, self-regulatory behaviors, learning experiences, and learning outcomes. This study extends previous research in three respects. First, it moves beyond isolated comparisons of individual AI design features by examining both the main and interaction effects of agent type and feedback style. Second, it adopts a multidimensional framework that connects the quality of AI-generated feedback with learners’ self-regulatory behaviors, subjective learning experiences, and short-term task improvement. Third, it combines quantitative and qualitative evidence to explain not only whether different feedback configurations are associated with different outcomes, but also how learners interpret, trust, regulate, and selectively use AI-generated feedback during authentic task completion. Accordingly, this study was guided by the following three research questions:

1. What are the main effects of agent type on feedback quality, self-regulatory behaviors, learning experiences, and learning outcomes?
2. What are the main effects of feedback style on feedback quality, self-regulatory behaviors, learning experiences, and learning outcomes?
3. Do interaction effects exist between agent type and feedback style?

## 2. Literature Review

### 2.1. Educational Agents in AI-Supported Learning Environments

The concept of agents originates from research in artificial intelligence, where agents are commonly defined as systems that perceive their environment and act upon it (Russell & Norvig, 2020). In educational contexts, educational agents are interactive computational systems embedded in learning environments that support learning through feedback, guidance, and dialogue (Baylor, 2011; Schroeder & Adesope, 2014). They are characterized by interactivity, goal orientation, and pedagogical functionality (Baylor & Ryu, 2003; Veletsianos & Russell, 2013). Earlier educational agents were often implemented as pedagogical agents, teachable agents, or intelligent tutoring systems (ITS). These systems typically relied on predefined scripts, rule-based interaction patterns, or structured domain models and were mainly effective in relatively well-structured learning domains (Graesser et al., 2005; Veletsianos & Russell, 2013). However, their capacity to support open-ended dialogue, dynamic content generation, and complex cognitive tasks was limited.

With the rapid development of large language models (LLMs), educational agents have increasingly incorporated natural language generation, contextual responsiveness, memory, planning, and tool use, making them more flexible for open-ended and dialogue-based learning tasks (L. Wang et al., 2024). In this study, LLM-based educational agents refer to AI systems configured for specific educational goals and used to provide feedback on students’ instructional design work through multi-turn natural language interaction (Steinert et al., 2024; Kumar et al., 2024). However, greater technical flexibility does not necessarily guarantee educational effectiveness, because the educational use of LLMs needs to be aligned with clear pedagogical goals and learning processes (Peláez-Sánchez et al., 2024; Yan et al., 2024). The value of such agents depends on how their knowledge sources, workflows, feedback strategies, and interaction functions are configured to support learners’ task goals and self-regulatory processes (Steiss et al., 2024; Wölfel et al., 2024).

Functionally, educational agents have expanded beyond simple question-answering tools to roles such as learning scaffolds, learning partners, teachable agents, and facilitators of dialogue. Prior research shows that these agents can support reflective writing, self-regulated learning, and learning-by-teaching, as well as enhance engagement and metacognitive awareness in STEM contexts (e.g., Chen et al., 2026; X. Luo et al., 2026; Neshaei et al., 2025; M. Wang et al., 2025). Overall, educational agents are shifting from information providers to more interactive participants in the learning process with the empowerment of LLMs. Prior reviews have shown the growing potential of AI applications to support teaching and learning in higher education, particularly in areas such as personalized support, assessment, and adaptive learning (Zawacki-Richter et al., 2019).

Importantly, prior research has shown that the design of educational agents, including the instructional roles they simulate, can influence learners’ perceptions, motivation, and learning outcomes (Baylor & Kim, 2005). More recent reviews further show that pedagogical agent research has expanded across diverse learning domains, agent roles, and design features, while continuing to emphasize the importance of agent design for learning, motivation, and affective outcomes (Dai et al., 2022; Schroeder et al., 2025). However, greater technical capability does not necessarily translate into better learning processes or outcomes. What may matter more is how educational agents are designed and configured in relation to learners’ needs. This makes agent type a particularly important design consideration in understanding how educational agents may support self-directed learning.

### 2.2. Agent Types: General-Purpose Versus Customized Agents

Differences in AI agent configuration may influence how learners perceive and use feedback. Research indicates that learners’ perceptions of an agent’s expertise and credibility may influence engagement with its output (Kotlyar & Krasman, 2025). In educational settings, AI agents can be broadly categorized based on system configuration into general-purpose and customized agents, a distinction this study conceptualizes as “agent type.”

General-purpose agents rely on the broad generalization capabilities of foundation models rather than domain-specific alignment. Designed for broad applicability, they primarily use interaction setup to define roles and feedback protocols. This design offers flexibility and ease of deployment, and their broad applicability may also allow them to respond flexibly across varied tasks and learner inputs. However, the absence of domain-specific grounding may reduce the precision and contextual relevance of the feedback they provide (Kasneci et al., 2023; Wölfel et al., 2024; Yan et al., 2024). When feedback is perceived as vague, generic, or insufficiently grounded, learners may question its credibility and become more cautious in using it (Henderson et al., 2025; Thomas et al., 2026). Thus, agent design is associated not only with differences in feedback content, but also with how learners perceive and engage with feedback during learning.

Customized agents, in contrast, are enhanced through the integration of domain-specific knowledge bases, expert personas, and structured pedagogical workflows. Such integration may support more adaptive and interpretable feedback generation by grounding responses in domain-specific knowledge and structured instructional support (Khosravi et al., 2022). It may also shape learners’ perception of the agent as a credible domain expert, influencing how they interpret and engage with feedback. Prior work has shown that learners’ trust in AI-powered educational tools is closely related to their willingness to adopt and engage with such systems, highlighting the importance of credibility and perceived expertise in feedback interpretation (Nazaretsky et al., 2025; Shin, 2021; L. Yang et al., 2025). In sum, these differences suggest that agent type may shape how feedback is perceived and used, with potential consequences for learners’ self-regulatory processes and learning outcomes.

### 2.3. Feedback Styles: Socratic Versus Directive Feedback

Feedback delivery shapes how learners engage with tasks and use feedback during learning. Prior research in educational psychology, AI-mediated learning, and online learning has shown that different feedback styles are associated with differences in cognitive engagement, motivation, self-regulatory responses, and learning outcomes (Alsaiari et al., 2026; Guo et al., 2014). In this study, two feedback styles, Socratic and directive, are examined. This selection was grounded in prior feedback research. Shute (2008) distinguished directive feedback, which tells learners what needs to be corrected, from facilitative feedback, which guides learners to revise through reflection. Socratic feedback can be viewed as a questioning-based form of facilitative feedback, whereas directive feedback represents explicit corrective guidance. Recent AI-feedback research similarly differentiates feedback according to information presentation and the extent to which learners are cognitively guided (Alsaiari et al., 2026). Together, they represent two distinct ways of supporting learners, one emphasizing reflection and self-correction and the other emphasizing explicit guidance and efficient revision.

The Socratic method originates from the dialogic practice associated with Socrates, a classical Greek philosopher whose questioning approach has had a lasting influence on Western traditions of dialogue, inquiry, and critical reasoning. Classical accounts of Socratic dialogue, particularly those associated with Plato and Xenophon, portray Socratic questioning as a dialogic process through which interlocutors are guided to examine assumptions, recognize contradictions, and refine their understanding (Vlastos, 1991). In educational contexts, the Socratic method is commonly understood as a questioning-based approach that supports guided inquiry rather than direct instruction. Its educational value lies in prompting learners to examine assumptions, clarify concepts, identify inconsistencies, and construct their own understanding through dialogue (Paul & Elder, 2006; Y.-T. C. Yang et al., 2005). In this study, Socratic feedback was therefore conceptualized as a feedback style that uses guiding questions and follow-up prompts to help learners diagnose problems and consider possible revisions, rather than simply providing corrective answers. This interpretation is consistent with research showing that Socratic questioning and dialogue can support critical thinking and inquiry-based reasoning in educational contexts (Mahoney et al., 2023; Oyler & Romanelli, 2014; Y.-T. C. Yang et al., 2005).

Building on this conceptual foundation, Socratic feedback employs guiding questions and graduated prompts to encourage learners to identify errors and construct solutions independently (Qiu et al., 2025; VanLehn, 2011; Wood et al., 1976). Consistent with constructivist principles, this approach may promote higher-order thinking and deeper conceptual understanding (Chi, 2009). By requiring learners to actively generate explanations and diagnose problems, Socratic feedback often involves greater cognitive effort during the learning process. Such engagement is associated with enhanced comprehension monitoring and reflective thinking (Xi et al., 2026), but it may also lead to slower task execution, particularly when learners lack sufficient support or when task complexity is high (van Gog & Paas, 2008; T. Wang et al., 2023).

Directive feedback is characterized by explicit corrective guidance and concrete solutions. By clarifying task requirements and reducing ambiguity, directive feedback may support more efficient task execution (Clark et al., 2011; Kester et al., 2006; Narciss & Huth, 2004). This style offers structured guidance and clear revision direction; however, when feedback is overly explicit, it may reduce opportunities for active knowledge construction (Koedinger & Aleven, 2007). By limiting opportunities for self-explanation, directive feedback is associated with greater reliance on externally provided solutions (Le et al., 2025) and may influence learners’ metacognitive strategy use (Zhang & Zhang, 2024). Under conditions of sustained reliance, it may also leave fewer opportunities for reflective processing and autonomous thinking (Poh & Lee, 2026).

### 2.4. Theoretical Perspectives on AI-Generated Feedback in SDL

#### 2.4.1. Zone of Proximal Development (ZPD) and Scaffolding

Self-directed learning (SDL) emphasizes learners’ active role in setting goals, managing the learning process, monitoring progress, and evaluating outcomes; however, this does not mean that learners can complete all learning tasks independently without external support (Garrison, 1997; Loyens et al., 2008; Panadero, 2017). This is particularly the case in complex and open-ended tasks such as instructional design, where learners need to consider multiple design elements, including instructional objectives, learner characteristics, activity sequences, resources, technology integration, and assessment methods. Therefore, external feedback is often needed to help learners identify gaps between their current performance and task standards and revise their work accordingly (Hattie & Timperley, 2007; Nicol & Macfarlane-Dick, 2006).

The theory of the ZPD provides a core perspective for understanding such support. According to this theory, learners can accomplish tasks beyond their current independent capability with support from teachers or more capable peers. Related scaffolding theory further emphasizes that effective support should be temporary, contextualized, targeted, and adjustable; its purpose is not to replace learners in task completion, but to help them gradually improve independent performance in complex cognitive activities (Puntambekar & Hubscher, 2005; Vygotsky, 1978; Wood et al., 1976). Thus, in AI-supported SDL, AI-generated feedback can be understood as a form of external scaffolding: it helps learners identify problems, interpret standards, regulate strategies, and continuously revise their work, thereby supporting their progression toward higher levels of performance (Azevedo & Hadwin, 2005; Carless & Boud, 2018; Tai et al., 2018).

In traditional instructional contexts, scaffolding within the ZPD is usually provided by teachers or more capable peers; in AI-supported learning environments, large language model (LLM)-based educational agents may partially take on similar scaffolding functions (Crompton & Burke, 2023; Kumar et al., 2024; Steinert et al., 2024). Because learners differ in their current understanding, task performance, and feedback needs, effective scaffolding requires dynamic adjustment to learners’ actual states. This means that support providers need not only to offer information, but also to continuously calibrate the intensity and form of support according to learner performance (Azevedo & Hadwin, 2005; van de Pol et al., 2010; Yan et al., 2024). LLM agents, with their capacity for multi-turn dialogue, contextual responsiveness, and task-specific feedback, have the potential to provide personalized, sustained, and dynamically adjustable support (Lan & Chen, 2024; Lim et al., 2024).

#### 2.4.2. Technology Acceptance Theory

Because the scaffolding support examined in this study is mediated by AI agents rather than provided solely by human teachers or peers, technology acceptance theory provides a useful perspective for understanding how learners experience and use AI-generated feedback. This perspective helps explain whether learners perceive AI feedback as easy to use, useful, and worth continued use (Chan & Hu, 2023; Davis, 1989). Perceived ease of use is particularly relevant because different forms of AI feedback may require learners to invest different degrees of information processing, judgment, and revision effort; therefore, cognitive load is also an important dimension for understanding AI feedback experience. Recent generative AI research also suggests that appropriate metacognitive support can improve learning experiences and, to some extent, influence learners’ cognitive load and technology acceptance in AI environments (Xu et al., 2025). Beyond initial acceptance, whether AI feedback can function effectively as scaffolding also depends on learners’ self-regulation during feedback dialogue, such as monitoring understanding, judging feedback value, selectively adopting suggestions, and revising their work (Carless & Boud, 2018; Tai et al., 2018; Winstone et al., 2017). In addition, perceived usefulness and sustained feedback use may be related to learners’ cognitive, behavioral, and emotional involvement; therefore, engagement provides an important complementary perspective for understanding the feedback-use process (Fredricks et al., 2004; Lim et al., 2024).

From this scaffolding perspective, the agent type and feedback style discussed earlier can be further understood as two key dimensions of AI feedback scaffolding design: agent type concerns the knowledge sources, task alignment, and related configurations of the scaffold, whereas feedback style concerns how the scaffold is expressed and delivered. In other words, the effect of AI feedback depends not only on agent type or feedback style alone, but on how a specific agent type and feedback style jointly constitute scaffolding that matches learners’ current needs (Puntambekar & Hubscher, 2005; van de Pol et al., 2010). Accordingly, this study examines how agent type and feedback style jointly shape AI-generated feedback quality, learners’ self-regulatory behaviors, learning experiences, and learning outcomes, operationalized as short-term task-specific improvement.

### 2.5. A Multidimensional Framework for Examining AI-Generated Feedback in SDL

Building on the above theoretical discussion, this study adopts a multidimensional analytical framework to examine how different AI-generated feedback designs shape learners’ feedback use and task engagement during authentic SDL task completion. The framework consists of four interrelated dimensions: feedback quality, self-regulatory behaviors, learning experiences, and learning outcomes. These dimensions should not be understood as an exhaustive model of self-directed learning itself; rather, they provide a structured analytical framework for examining AI-supported feedback use in this study. Feedback quality represents the characteristics of the support provided, particularly whether feedback is accurate, specific, and aligned with task goals. Self-regulatory behaviors reflect learners’ monitoring, evaluation, and revision during task engagement, and therefore correspond most directly to the regulatory processes central to SDL. Learning experiences reflect how learners perceive and respond to the feedback process, including technology acceptance, engagement, critical thinking openness, reflective skepticism, and cognitive load, all of which may shape their willingness and ability to engage with feedback. Learning outcomes refer to short-term task-specific improvement in this study, measured by the extent to which learners improved the quality of their instructional design drafts after engaging with AI-generated feedback. This measure reflects immediate task performance improvement rather than long-term learning, transfer, or general SDL development.

## 3. Methods

### 3.1. Ethics Statement

The research protocol for this study received an exemption from the Central China Normal University Institutional Review Board (Approval No. CCNU-IRB-202503076b). The exemption was granted because the study involved adult postgraduate students, was conducted in a regular educational setting, and posed no more than minimal risk to participants. Prior to data collection, participants were informed about the purpose of the study, the research procedures, and data privacy protections, and informed consent was obtained from all participants. Participation was entirely voluntary, and participants had the right to withdraw at any time or skip specific questions if they wished. All data were anonymized prior to analysis and coded in a way that prevented identification of individual participants, thereby ensuring confidentiality and data security throughout the research process.

### 3.2. Research Context and Participants

This study was conducted during the fall semester of 2025 within a graduate-level course titled Educational Technology Theory and Practice at a research-oriented university in China. The research was embedded in the regular course assignments, and the corresponding author served as the course instructor. All participants completed identical learning tasks and received standardized instructional materials to minimize instructional variability. Initially, 54 master’s students participated. Three participants were excluded due to incomplete Learning Experience Questionnaire (LEQ) data, resulting in a final analytical sample of 51 participants. The final sample included 46 female students (90.2%) and 5 male students (9.8%), indicating a gender imbalance that is further addressed in the Limitations Section.

The cohort size was considered appropriate for the course-embedded mixed-methods experimental design of this study. Sample size justification should be aligned with the research purpose, design features, available population, and inferential goals rather than determined by a universal numerical threshold (Lakens, 2022). In this study, the final sample of 51 postgraduate students represented the available course cohort after excluding incomplete responses. The mixed factorial design also provided repeated-measures data for examining within-participant differences across the two feedback-style conditions, which can improve statistical efficiency by reducing error variance associated with between-participant differences (Maxwell et al., 2017). In addition, multiple sources of process and outcome data were used to contextualize and explain the quantitative findings, consistent with sampling considerations in mixed-methods research (Onwuegbuzie & Collins, 2007). As a supplementary check, a sensitivity power analysis using G*Power 3.1 (Faul et al., 2007) indicated that the final sample size was sufficient to detect effects of approximately f = 0.20 or larger.

### 3.3. Experimental Conditions

All AI agents were developed using the Coze platform (https://www.coze.cn, accessed on 26 June 2026). To control for possible confounding effects of the technical platform and underlying model architecture, all agents were built on the same LLM (Kimi-32K) with identical interaction interfaces, response latency controls, and basic dialogue parameters. The four experimental conditions are detailed in Table 1.

Customized agents used two specialized knowledge bases alongside prompts: the “Instructional Design Textbook,” covering key design principles, and the “Instructional Design Evaluation Rubric,” with a seven-dimensional evaluation framework. The detailed rubric is provided in Supplementary Table S1. In the initialization phase, they called the “Textbook” through Recall Knowledge, and in the feedback phase, they used the “Rubric” to structure feedback. These agents also followed a defined workflow that dictated the order of the seven dimensions (e.g., completeness → systematization → teaching strategies), with specific entry and termination conditions, ensuring structured and consistent feedback generation across interactions. In contrast, general-purpose agents relied only on prompts for feedback generation, without external knowledge or workflow. Their feedback followed a similar seven-dimension structure, but progression was determined by the model’s context, not predefined workflow steps.

Socratic feedback was operationalized as a sequence of guiding questions and follow-up prompts that asked students to examine assumptions, identify possible inconsistencies, and consider alternative revisions in their instructional designs. It encouraged students to identify issues through question-based prompts, such as “Have you considered …?” and “Could this be expressed more clearly?”, rather than offering direct corrections. Feedback focused on one specific issue per dimension, with follow-up prompts if answers were unclear, offering minimal examples when needed. All feedback was anchored to the student’s submission, avoiding generalized advice. Directive feedback directly pointed out deficiencies in the student’s work and provided clear, actionable suggestions. Each round addressed one specific issue and gave improvement directions, with examples provided if responses were unclear. Feedback was also anchored to the student’s text and avoided vague questions, ensuring efficiency and applicability. Representative one-turn feedback excerpts from each of the four experimental conditions are provided in Supplementary Table S2 to illustrate how agent type and feedback style were operationalized.

### 3.4. Research Design and Procedure

This study employed a 2 × 2 mixed factorial design, with agent type (customized vs. general-purpose) as a between-subjects factor and feedback style (Socratic vs. directive) as a within-subjects factor. The customized agent group included 27 participants, and the general-purpose agent group included 24 participants. The experiment was conducted over two consecutive weeks, with each participant completing one instructional design task per week. All participants received Socratic feedback in Week 1 and directive feedback in Week 2. This design yielded four condition combinations across the two agent types and two feedback styles: customized + Socratic, general-purpose + Socratic, customized + directive, and general-purpose + directive. Because the study was conducted in an authentic course context, the feedback styles were administered in a fixed sequence rather than counterbalanced. This approach provided a feasible and pedagogically coherent way to implement the intervention within regular course activities while maintaining consistency across participants. Although this design may still involve some order-related influence, it was considered the most appropriate arrangement under the instructional conditions of the study.

To minimize the influence of potential confounding factors, the two instructional design tasks were embedded in regular course assignments and were designed to maintain a high degree of consistency in structural framework, difficulty level, and assessment criteria. They intentionally differed in teaching context and technological theme, in order to balance task equivalence with contextual novelty and to reduce the potential influence of task familiarity on the experimental results. Specifically, in Week 1, students designed a lesson on Artificial Intelligence and Educational Applications; in Week 2, they designed a geography project-based lesson on sustainable future cities supported by virtual reality technology.

Both tasks required students to independently complete a full instructional design plan, including learning objectives, learner analysis, learning activity design, resources and environment arrangements, and assessment methods. Both tasks involved higher-order cognitive operations: students needed not only to understand basic instructional design principles, but also to integrate a specific technology, either AI or VR, into a coherent instructional process. Thus, the two tasks reflected comparable task complexity and knowledge integration requirements. At the same time, differences in educational level and technology type helped reduce familiarity effects in the second week while maintaining cross-task comparability. A detailed comparison of the two tasks is presented in Table 2.

**Figure 1:**
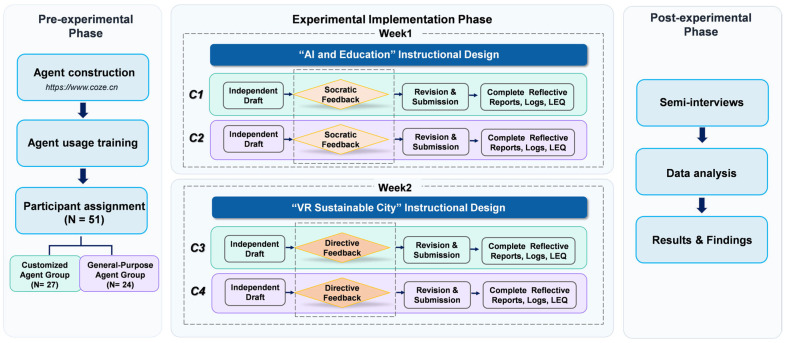
The experimental procedure.

For each task, participants first completed an independent draft, then interacted asynchronously with the designated AI agent to receive feedback and revise their instructional design. After completing each task, participants wrote a reflective report, saved their AI interaction logs, and completed the Learning Experience Questionnaire (LEQ) to evaluate their experience with AI-assisted feedback. All materials (revised instructional designs, reflective reports, and interaction logs) were submitted and managed through the Xiaoya platform. An overview of the experimental procedure is shown in Figure 1.

### 3.5. Data Collection

This study employed multiple data sources to measure four key dependent variables: feedback quality, self-regulatory behaviors, learning experiences, and learning outcomes.

First, feedback quality was assessed through multidimensional coding of the complete human–AI interaction logs. The coding framework was adapted from Steiss et al. (2024) on the quality of generative AI feedback and further contextualized to fit the instructional design task. Five dimensions were included: criteria-based relevance, specificity, accuracy, prioritization of essential features, and supportive tone. Each dimension was rated on a five-point scale (1–5), with clearly defined behavioral anchors to enhance coding consistency and interpretability. Detailed operational definitions and scoring criteria are provided in Supplementary Table S3. Coding was conducted at the level of each student’s complete interaction log. Two trained coders independently reviewed the full set of AI-generated feedback within each interaction and assigned holistic ratings across the five dimensions. Each student received one coded record per task, resulting in two feedback quality scores corresponding to the Socratic and directive feedback conditions. Inter-rater reliability was satisfactory (intraclass correlation coefficient [ICC] = 0.758), and the mean score of the two coders’ ratings was used for analysis. Representative high and low scoring excerpts for the five dimensions of feedback quality are provided in Supplementary Table S4.

Second, self-regulatory behaviors were also coded based on the complete interaction logs. Drawing on self-regulated learning theories (Winne & Hadwin, 1998; Zimmerman, 2002), student behaviors were operationalized into three dimensions: task orientation, comprehension monitoring, and feedback regulation. Each dimension was rated on a five-point scale (1–5), and detailed coding criteria are provided in Supplementary Table S5. Like feedback quality, coding was conducted at the level of each student’s complete interaction process. Two coders independently evaluated students’ questioning, responses, and adaptive behaviors throughout the interaction and assigned holistic ratings for each dimension. The coding procedure was identical to that used for feedback quality, and the mean of the two coders’ ratings was used as the final score. Inter-rater reliability was high (ICC = 0.868). Representative high and low scoring excerpts for the three dimensions of self-regulatory behaviors are provided in Supplementary Table S6.

Third, learning experiences were measured using a questionnaire assessing students’ subjective perceptions of different agent types and feedback styles across four domains: technology acceptance, learning engagement, critical thinking disposition, and cognitive load. Technology acceptance was adapted from the Technology Acceptance Model (Davis, 1989), including three subdimensions: perceived ease of use, perceived usefulness, and intention to use (16 items). Learning engagement was measured based on the three-dimensional engagement framework discussed in the literature review, including cognitive, behavioral, and emotional engagement (15 items). Critical thinking disposition was measured using the Critical Thinking Disposition Scale (Sosu, 2013), including two subdimensions: critical thinking openness and reflective skepticism (11 items). Cognitive load was adapted from the extraneous cognitive load subscale (Leppink et al., 2013) consisting of four items assessing the additional cognitive effort required to process feedback. All items were rated on a five-point Likert scale (1 = strongly disagree, 5 = strongly agree), and the questionnaire was administered after each task. Reliability analysis indicated good internal consistency across all dimensions (Cronbach’s α = 0.725–0.913). The full questionnaire and subscale structure are provided in Supplementary Table S7.

Finally, learning outcomes were operationalized as short-term task-specific improvement and quantified using gain scores, calculated as the difference between revised and initial instructional design drafts (gain score = revised score − initial score). In each task, students first submitted an initial draft independently and then revised it after interpreting, evaluating, and selectively incorporating AI-generated feedback. Thus, the gain score captured immediate improvement in instructional design performance after feedback use. This measure was task-specific and closely linked to the feedback design, making it suitable for examining differences across experimental conditions. This approach also controlled individual baseline differences and more accurately captured task improvement under different experimental conditions. Both initial and revised drafts were evaluated using a unified instructional design rubric developed based on established theoretical and practical standards in the field. The rubric comprised seven dimensions with a total weight of 100% (see Supplementary Table S1). All assignments were scored on a 100-point scale by two trained raters with expertise in educational technology. To enhance scoring consistency, both raters used the same rubric and scoring criteria throughout the evaluation process. Any discrepancies were resolved through discussion until consensus was reached, and the final score was based on the agreed evaluation.

### 3.6. Data Analysis

We adopted a mixed-methods analysis strategy. For the quantitative data, descriptive statistics were first calculated. Then, a 2 × 2 mixed-design ANOVA was conducted using SPSS 24.0 to examine the main and interaction effects of agent type and feedback style on all dependent variables. Although the Shapiro–Wilk test indicated deviations from normality for several variables, the mixed-design ANOVA was retained. This decision was based on evidence that ANOVA is robust to moderate violations of normality, particularly when group sizes are comparable and the design is approximately balanced (Blanca et al., 2017; Schmider et al., 2010). In addition, effect sizes were reported to provide a more comprehensive interpretation of the results.

For the qualitative data, reflection reports were analyzed following the thematic analysis procedures proposed by Braun and Clarke (2006). All textual materials were first read repeatedly to achieve familiarity with the content and to identify preliminary patterns related to the research questions. The first author then conducted initial inductive coding to capture meaningful units concerning participants’ perceptions, cognitive processes, and feedback utilization (Saldaña, 2009). The initial codes were refined into coding nodes, and similar nodes were grouped into broader categories through constant comparison. Related categories were then synthesized into overarching themes to interpret learners’ engagement with AI-generated feedback. To enhance transparency and trustworthiness, the coding nodes, category structure, and theme interpretation were reviewed by the research team and refined through iterative discussion until consensus was reached. The final thematic structure is reported in Section 4.3 and Appendix A.

## 4. Results

### 4.1. Descriptive Statistics

Descriptive statistics for all dependent variables across the four condition combinations are presented in Table 3. Overall, customized agents showed descriptively higher scores on feedback accuracy and specificity than general-purpose agents. Differences in self-regulatory behaviors were more visible for comprehension monitoring than for task orientation and feedback regulation. Most dimensions of learning experience varied only slightly across conditions, with cognitive load showing relatively clearer differences. In terms of learning outcomes, gain scores were descriptively higher under Socratic feedback, whereas under directive feedback the customized agent showed higher gains than the general-purpose agent. Inferential analyses using mixed-design ANOVAs are reported in the following sections.

### 4.2. Main Effects and Interaction Effects

#### 4.2.1. Feedback Quality

Mixed-design ANOVA results showed a significant main effect of agent type on feedback accuracy (*η*^2^*p* = 0.368) and specificity (*η*^2^*p* = 0.176), indicating large and moderate effects. Feedback style also yielded significant main effects on the prioritization of essential features (*η*^2^*p* = 0.178) and specificity (*η*^2^*p* = 0.129). No significant interaction effects between agent type and feedback style were found across feedback quality dimensions. Detailed results are presented in Table 4.

#### 4.2.2. Self-Regulatory Behaviors

Mixed-design ANOVAs were conducted to examine the effects of agent type and feedback style on self-regulatory behaviors. No significant main effect of agent type was found for task orientation, comprehension monitoring, or feedback regulation. In contrast, feedback style showed significant main effects on task orientation (*η*^2^*p* = 0.226) and comprehension monitoring (*η*^2^*p* = 0.339), indicating moderate to large effects. Descriptively, scores for both task orientation and comprehension monitoring were higher under Socratic feedback than under directive feedback across the two agent types. No significant main effect of feedback style was observed for feedback regulation. However, a significant interaction effect emerged for feedback regulation *(η*^2^*p* = 0.078), indicating that the pattern of feedback regulation varied across the combinations of agent type and feedback style. Detailed statistics are presented in Table 5.

#### 4.2.3. Learning Experiences

Mixed-design ANOVAs were conducted to examine the effects of agent type and feedback style on learning experiences. As seen in Table 6, results showed no significant main effects of agent type on perceived ease of use, perceived usefulness, intention to use, cognitive engagement, behavioral engagement, emotional engagement, critical thinking openness, reflective skepticism, or cognitive load. Likewise, no significant interaction effects between agent type and feedback style were found across these dimensions. Feedback style showed a significant main effect only on cognitive load (*η*^2^*p* = 0.113), indicating a moderate effect. Descriptively, cognitive load scores were higher under directive feedback than under Socratic feedback across both agent types. For the other dimensions of learning experience, the descriptive differences across conditions were relatively small.

#### 4.2.4. Learning Outcomes

**Figure 2:**
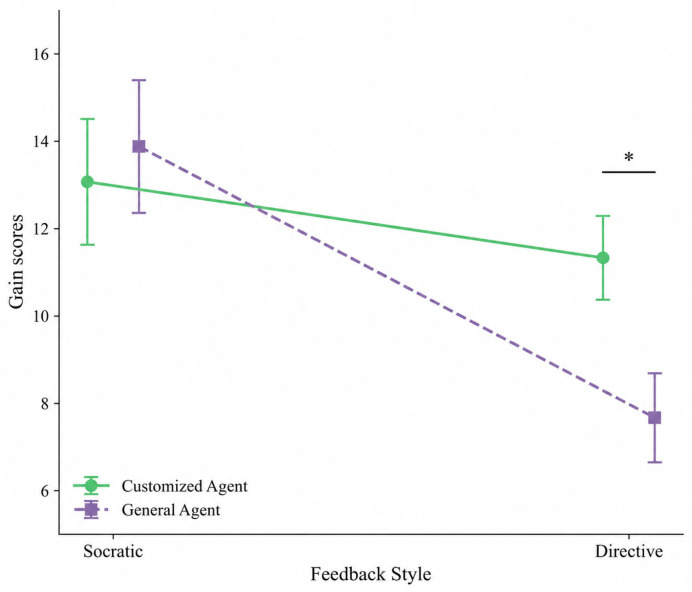
Interaction between agent type and feedback style on short-term task improvement. The figure shows mean gain scores from the initial to revised instructional design drafts across the customized and general-purpose agent groups under Socratic and directive feedback conditions. Error bars represent standard errors. The asterisk indicates a significant simple effect of agent type under directive feedback (*p* < 0.05), whereas no significant difference was observed between agent types under Socratic feedback.

Mixed-design ANOVA revealed a significant main effect of feedback style on learning outcomes (F_(1,49)_ = 15.218, *p* < 0.001, *η*^2^*p* = 0.237), with gain scores overall being higher under Socratic feedback than under directive feedback. The main effect of agent type was not statistically significant (F_(1,49)_ = 0.961, *p* = 0.332, *η*^2^*p* = 0.019). A significant interaction effect between agent type and feedback style was also found (F_(1,49)_ = 4.807, *p* = 0.033, *η*^2^*p* = 0.089). As shown in Figure 2, simple-effects analysis indicated that under Socratic feedback, there was no significant difference in gain scores between the customized agent and the general-purpose agent (F_(1,49)_ = 0.147, *p* = 0.703, *η*^2^*p* = 0.003). Under directive feedback, however, the customized agent produced significantly higher gain scores than the general-purpose agent (F_(1,49)_ = 6.843, *p* = 0.012, *η*^2^*p* = 0.123). These results indicate that gain scores varied by the combination of agent type and feedback style.

### 4.3. Qualitative Findings Based on Reflection Report and Interview

To further understand how learners perceived and engaged with AI-generated feedback, a qualitative analysis was conducted based on reflection reports and interview data. The coding process yielded 16 nodes across 10 categories, which were organized into three overarching themes. Appendix A presents the thematic structure developed in this study.

#### 4.3.1. Theme 1: Socratic Feedback Supported Reflective Monitoring, Whereas Directive Feedback Was Associated with Cognitive Overload

Qualitative analysis suggests that feedback style was associated with different patterns of cognitive engagement during feedback use. Socratic feedback was characterized by dialogic prompting, which appeared to support reflective monitoring. Follow-up questioning was observed exclusively in the Socratic feedback groups (C1 and C2), with no instances identified in the directive feedback groups. Students reported that such questioning encouraged them to examine the logic of their instructional design more closely. For example, one student noted, “It constantly asks follow-up questions that guide me to think about areas worth improving in my instructional design.” Students described this process as a shift from surface-level revision to deeper reasoning, as one student stated during an interview: “It guides me to think instead of telling me the answer directly.” However, reflective engagement was not limited to Socratic feedback. Codes related to deep logic reflection were observed across all groups, with relatively higher frequencies in the directive feedback groups. This indicates that while Socratic feedback supports reflective monitoring through questioning, deeper reflection is not exclusively associated with this feedback style.

In contrast, directive feedback was more frequently associated with perceptions of overload and resistance. Codes related to overload and resistance were more prevalent in the directive feedback groups (C3 and C4). Students described experiencing difficulty managing multiple suggestions simultaneously. For instance, one student reflected, “This pressure arises from information overload. There is too much information, and I have to spend time digesting all these suggestions.” Another noted that continuous identification of issues led to frustration: “After you finish revising one point, there will always be problems, and it will always tell me where there are problems that need to be revised.” These accounts suggest that directive feedback may impose higher implementation-related cognitive demands, which can lead to feelings of overload and, in some cases, resistance to further interaction.

#### 4.3.2. Theme 2: Learners Selectively Used AI-Generated Feedback While Maintaining Agency

When processing AI-generated suggestions, learners exhibited strategic filtering tendencies, suggesting the maintenance of pedagogical agency. Of the 114 codes related to critical rejection of feedback, the Socratic feedback groups showed relatively higher frequencies. The interaction patterns observed in feedback regulation behavior may reflect learners’ agency when responding to AI-generated suggestions. Reflection reports documented students’ clear rejection of low-quality feedback: “Answers such as ‘making teaching priorities more prominent’ and ‘the teaching process more in line with students’ learning laws’ were not adopted because no specific examples were given.” This behavior was described in an interview by one student as a process of identification and filtering: “Some parts, I can clearly feel, do not meet my requirements, so I can identify them.”

Meanwhile, the adoption of high-quality feedback was positioned as a means to optimize existing designs rather than substitute for thinking. Of the 446 codes related to complete feedback adoption, the C3 group accounted for 122 instances, demonstrating highly task-oriented behavior. Reflection reports stated: “I will continue to use AI as a thinking partner but will maintain my subjectivity as an educator.” One student also expressed this pragmatic feedback filtering logic during an interview: “In terms of cognitive inspiration, the first scaffold is useful, but it might not be as useful to me as the second one.” This suggests that learners maintained cognitive control to ensure that AI-supported revisions aligned with their instructional logic. These data suggest that learners did not adopt AI-generated feedback passively; instead, they actively filtered and selectively incorporated suggestions into their revisions.

#### 4.3.3. Theme 3: Customized Agents Were More Often Associated with Trust and Perceived Credibility

Codes related to positive feedback regarding clarity and operability were more prevalent in the customized groups (C1 and C3). As reported in Section 4.2.1, customized agents significantly outperformed general-purpose agents in feedback accuracy and specificity—two key dimensions that theoretically underpin learners’ perceptions of source credibility. Reflection reports documented this trust based on accuracy: “I understand the agent’s feedback. It is very clear and I can understand it all.” One student also confirmed the professional advantage of customized agents during an interview: “This agent, in terms of outputting certain content, feels more professional than what I would write myself, and it is also more appropriate.” These qualitative accounts suggest that clearer and more contextually aligned feedback from customized agents was often associated with stronger perceptions of trust.

In contrast, general-purpose agents without a professional knowledge base often face challenges in establishing trust due to contextual disconnection. Of the 68 codes related to negative feedback about lack of contextual anchoring, C4 was dominant. One student reflected during an interview: “In the first week, I felt that what it gave was not that appropriate.” When customized agents demonstrated deep contextual understanding, students’ perception of their role shifted. Reflection reports stated: “These suggestions indeed addressed the pain points of the original plan, namely vague theory and blurred evaluation.” This cognitively driven trust, based on content accuracy, was associated with a shift in how learners perceived the role of AI. This aspect of trust was reflected in the reports: “The knowledge extraction for the questions it answers should be more appropriate.” Together with the quantitative evidence of enhanced accuracy, these qualitative findings suggest that perceived feedback quality may be associated with learners’ trust in the agent. Such perceptions may be associated with learners’ engagement patterns and may help explain differences in learning outcomes observed under directive feedback.

## 5. Discussion

### 5.1. Effects of Agent Type

Agent type showed significant effects primarily on the quality of AI-generated feedback. Customized agents outperformed general-purpose agents in feedback accuracy and specificity, suggesting that domain-specific configuration enhanced the informational quality of the support provided. This advantage likely stemmed from two related features. First, customized agents drew on domain-specific knowledge bases, including instructional design theories, evaluation criteria, and example resources, which may have improved the precision and contextual fit of the feedback (Alsaiari et al., 2026; Khosravi et al., 2022). This interpretation is also consistent with prior concerns that AI systems without sufficient domain-specific grounding may produce feedback that is less precise or less contextually aligned (Kasneci et al., 2023; Wölfel et al., 2024; Yan et al., 2024). Second, customized agents followed a structured process based on explicit evaluation dimensions, which may have supported more systematic coverage of key aspects of instructional design while reducing vague or generic comments (Steiss et al., 2024). This explanation is also supported by the qualitative findings, in which learners described customized agents as clearer, more professional, and better aligned with task requirements. From a scaffolding perspective, this suggests that customized agents provided more task-aligned support by grounding feedback in domain-specific knowledge, evaluation criteria, and structured workflows. These findings extend previous research by showing that the primary advantage of customization may lie in improving the generation and grounding of feedback, rather than producing a general advantage across all aspects of learning.

However, agent type did not significantly affect self-regulatory behaviors, learning experiences, or learning outcomes. This pattern suggests that greater domain customization and higher feedback quality do not necessarily translate into broader educational benefits. In these open-ended instructional design tasks, learners still needed to interpret suggestions, make independent judgments, and integrate feedback into their own design logic. As a result, improvements in feedback accuracy and specificity did not necessarily translate into differences in regulatory behaviors, subjective experience, or task improvement. The findings therefore distinguish between the quality of feedback generated by an AI system and the learning consequences of using that feedback. More accurate and specific feedback may provide stronger informational support, but its educational value still depends on whether learners understand, evaluate, and productively incorporate it into their work. This interpretation is consistent with prior work emphasizing that feedback supports learning only when learners actively interpret and use it in relation to their own goals (Hattie & Timperley, 2007; Winstone et al., 2017).

The absence of significant differences in learning experiences between agent types is also noteworthy. Although the qualitative findings suggest that clearer and more contextually aligned feedback from customized agents may have contributed to stronger perceptions of professionalism and credibility, these differences did not extend to broader self-reported learning experiences. This pattern is consistent with prior research showing that learners’ perceptions of AI tools are often shaped more by the interaction process and perceived usefulness of the system than by differences in underlying system design (Labadze et al., 2023), and it also aligns with research highlighting the importance of credibility and trust in learners’ engagement with AI-supported tools (Nazaretsky et al., 2025; Shin, 2021). Taken together, these results suggest that customization may improve the conditions under which high-quality and credible feedback can be produced, rather than serving as a sufficient condition for improved self-regulation, learning experience, or performance. Its broader educational value may depend on how learners engage with the feedback and on the feedback style with which the customized agent is paired.

### 5.2. Effects of Feedback Style

Feedback style appears to matter because it organizes learners’ cognitive work in different ways. However, because the two feedback styles were implemented in a fixed sequence, the observed differences should be interpreted as condition-related patterns, not definitive causal effects of feedback style alone. In the present study, directive feedback was more closely associated with feedback quality dimensions related to specificity and prioritization, whereas Socratic feedback was more closely associated with self-regulatory behaviors, particularly task orientation and comprehension monitoring. Rather than suggesting that one style is generally superior, this pattern indicates that the two feedback styles may represent different forms of scaffolding.

Directive feedback appeared to support revision by reducing ambiguity and making improvement targets more explicit. This interpretation is consistent with prior work showing that structured and explicit feedback can facilitate efficient task execution and revision (Clark et al., 2011; Kester et al., 2006; Narciss & Huth, 2004). At the same time, the literature on feedback and metacognition has cautioned that highly explicit guidance may reduce opportunities for self-explanation and active knowledge construction when learners rely too directly on externally provided solutions (Koedinger & Aleven, 2007; Le et al., 2025; Poh & Lee, 2026; Zhang & Zhang, 2024). This tension was also visible in the qualitative data, where directive feedback was perceived as clearer and more actionable, but also more cognitively demanding. Thus, the explicitness of directive feedback may provide immediate procedural support while simultaneously transferring to learners the task of evaluating, coordinating, and implementing multiple concrete suggestions.

Socratic feedback, by contrast, was associated with learners’ regulatory involvement by prompting them to interpret questions, examine their own reasoning, and monitor their understanding during revision. This interpretation aligns with prior work suggesting that questioning-based support promotes reflective thinking, self-explanation, and metacognitive engagement (Chi, 2009; VanLehn, 2011; Winne & Hadwin, 1998; Xi et al., 2026; Zimmerman, 2002). In self-directed learning contexts, this distinction is especially important because effective support should not only improve immediate performance but also sustain learners’ active role in evaluating and revising their own work. The present findings extend this line of research by suggesting that the reflective value of Socratic questioning is also observable in human–AI feedback interactions, where learners must remain active participants rather than merely recipients of AI-generated solutions.

The finding that directive feedback was associated with higher cognitive load further clarifies this trade-off. From a cognitive load perspective, dense and highly explicit feedback may increase processing demands when learners must evaluate and implement multiple suggestions within limited working memory resources (Paas & van Merriënboer, 2020; Skulmowski & Xu, 2022; Sweller et al., 2011). Importantly, this result suggests that direct guidance does not necessarily reduce all forms of cognitive demand. Although directive feedback may reduce the effort required to identify problems or generate possible solutions, it may increase implementation-related demands because learners must process, prioritize, reconcile, and apply multiple recommendations to their work. This distinction between solution-generation demands and feedback-implementation demands provides a more nuanced explanation of why explicit feedback may be both actionable and cognitively burdensome. Taken together, Socratic feedback was more closely associated with reflective monitoring and metacognitive engagement, whereas directive feedback appeared to provide more explicit revision guidance and was associated with greater cognitive processing demands.

At the same time, the non-significant findings for most learning-experience dimensions should also be noted. Apart from cognitive load, no significant differences were observed between the two feedback-style conditions in perceived ease of use, perceived usefulness, intention to use, cognitive engagement, behavioral engagement, emotional engagement, critical thinking openness, or reflective skepticism. One possible explanation is that both feedback styles were delivered through the same platform, underlying model, and interaction interface; therefore, learners may have perceived similar usability across conditions (Davis, 1989; Chan & Hu, 2023). In addition, because the intervention was embedded in two short-term course tasks, learners may not have detected subtle differences in the usefulness of the two feedback styles (Winstone et al., 2017). The relatively high scores for critical thinking openness and reflective skepticism also suggest that these postgraduate learners generally maintained a critical stance toward AI-generated feedback, which may have reduced observable differences between conditions (Chan & Hu, 2023; Yan et al., 2024). These non-significant findings further indicate that changing feedback style alone may not be sufficient to alter learners’ broader perceptions of AI-supported learning, particularly when the technological environment, task context, and underlying model remain constant.

### 5.3. Interaction Effects of Agent Type and Feedback Style

The interaction between agent type and feedback style is a notable finding of this study because it indicates that their influence on learning outcomes should be understood in combination rather than in isolation. This finding extends previous research that has typically examined agent configuration or feedback style as separate design features. It suggests that the educational value of an AI feedback system may depend not only on whether the agent is customized or general-purpose, but also on how its feedback is delivered and processed by learners. In this sense, the effects of AI-generated feedback appear to be conditional and configurational rather than simply additive. Although customized agents did not show an overall advantage in learning outcomes, their advantage emerged under directive feedback but not under Socratic feedback.

One plausible explanation is that directive feedback relies more heavily on the immediate usability of feedback. When learners revise their work based on explicit suggestions, the extent to which feedback supports revision may depend more strongly on whether those suggestions are accurate, specific, and aligned with the task, which is consistent with prior research on actionable feedback and revision (Hattie & Timperley, 2007; Narciss, 2013; Shute, 2008). Under this condition, the higher accuracy and specificity of customized agents may be more readily translated into concrete revisions because learners are expected to act directly on the suggestions provided. From a scaffolding perspective, this interaction suggests that AI-generated feedback should be understood as a configuration of support rather than as the effect of a single design feature. In this configuration, agent type shapes how the scaffold is grounded, whereas feedback style shapes how the scaffold is delivered and used during revision. The interaction therefore connects two distinct aspects of AI feedback design: the informational grounding of the support and the cognitive process through which learners engage with that support.

By contrast, under Socratic feedback, no significant difference in gain scores was found between customized and general-purpose agents. This suggests that questioning-based support may reduce learners’ dependence on the precision of the external feedback source. Because Socratic feedback requires learners to interpret prompts, generate explanations, and construct their own revisions, learners may compensate for limitations in feedback quality through self-explanation and reasoning, consistent with prior work on generative processing and guided questioning (Chi et al., 1994; King, 1992). In other words, when feedback functions primarily as a prompt for learner-generated reasoning, the quality of learners’ own interpretive and reflective activity may partly offset differences in the agent’s domain-specific configuration. This interpretation is also consistent with the qualitative findings, which showed that learners regulated and applied AI-generated feedback differently across the combinations of agent type and feedback style. Socratic feedback appeared to place greater emphasis on learner-generated reflection, whereas directive feedback supported more direct execution of revisions.

The theoretical significance of this interaction lies in showing that customization is not universally advantageous. Its contribution depends on the cognitive demands created by the feedback style and on the extent to which learners rely directly on the content supplied by the agent. Customized agents may therefore be particularly valuable when learners are expected to act directly on explicit suggestions, whereas their advantage may be less pronounced when learners generate revisions through questioning-based reflection. This conditional account moves beyond asking which type of agent or feedback style is generally more effective and instead highlights when and why particular combinations may support self-directed learning.

### 5.4. Theoretical Contributions and Practical Implications

Theoretically, this study extends research on AI-generated feedback in self-directed learning in three related ways. First, it moves beyond examining agent type and feedback style as isolated design features by showing that their associations with learning depend partly on how they are combined. The interaction observed for short-term task improvement supports a conditional and configurational view of AI feedback design: agent type shapes how feedback is grounded in domain knowledge and task criteria, whereas feedback style shapes how learners cognitively engage with and act on that feedback. Second, the findings distinguish feedback quality from the broader educational consequences of feedback use, showing that greater accuracy and specificity do not automatically translate into stronger self-regulation, learning experiences, or performance. Third, the different patterns associated with Socratic and directive feedback suggest that feedback styles may distribute cognitive and regulatory responsibility differently between the AI agent and the learner. Together, these findings highlight the significance of examining AI-generated feedback as an integrated design configuration in which feedback quality, delivery style, and learners’ active interpretation jointly shape AI-supported learning.

Building on these theoretical insights, the findings also offer practical implications for learners, teachers, and AI agent designers. Learners should use AI-generated feedback strategically rather than passively. Directive feedback may provide explicit revision guidance, whereas Socratic feedback may support reflection and regulation in SDL. Teachers should choose feedback styles according to instructional goals. When the goal is to help students revise more directly and improve short-term task performance, directive feedback may be useful, particularly when it is provided by a customized agent. When the goal is to support monitoring, reflection, and self-regulation in the feedback process, Socratic feedback may be more appropriate. AI agent designers should align agent design with feedback purpose. To support directive and actionable feedback, they should strengthen domain-specific knowledge bases and structured workflows to improve feedback accuracy and specificity. To support reflection and self-monitoring, they should use prompting strategies that encourage learners to question, explain, and evaluate their own work.

### 5.5. Limitations and Future Research

Several limitations should be considered when interpreting the findings of this study. First, the sample consisted of postgraduate students from a single university and disciplinary context, with an imbalanced gender distribution, which may limit the generalizability of the findings. Future studies could include more diverse learner populations across institutions, disciplines, and levels of expertise. Second, task equivalence was established mainly through parallel task design and a shared scoring rubric rather than through independent baseline difficulty tests. Future studies could include independent difficulty assessments to further validate task comparability. Third, the two feedback styles were implemented in a fixed sequence within an authentic course context. Although this provided ecological validity, order-related influences cannot be fully ruled out. Future studies could adopt counterbalanced or randomized designs to isolate feedback-style effects more precisely. Fourth, learners’ experiences were measured mainly through self-report questionnaires, which may not fully capture the real-time cognitive and emotional processes involved in AI-supported feedback use. Future research could incorporate multimodal data, such as behavioral traces, physiological measures, or process-based learning analytics, to provide more fine-grained evidence. Finally, learning outcomes in this study were operationalized narrowly as short-term task improvement, measured by gains in instructional design performance from the initial to revised drafts. Although this measure captured task improvement, it may not fully reflect deeper conceptual learning, longer-term transfer, or general SDL development. A clearer distinction between immediate performance gains and broader learning outcomes would strengthen future evaluations of AI-generated feedback.

## 6. Conclusions

This study examined how agent type and feedback style were associated with learners’ engagement with AI-generated feedback in self-directed learning. The findings suggest that customized agents were mainly associated with higher feedback quality, particularly in terms of accuracy and specificity, whereas feedback style was more strongly associated with differences in self-regulatory behaviors, cognitive load, and short-term task improvement. Socratic feedback was associated with stronger comprehension monitoring, whereas directive feedback appeared to provide more direct revision guidance and was associated with higher cognitive load. A significant interaction effect further suggested that the advantage of customized agents in short-term task improvement emerged under directive feedback but not under Socratic feedback. Taken together, these findings suggest that AI-generated feedback is best understood as an integrated design in which agent type, feedback style, and learners’ active feedback use operate together. Customization may strengthen feedback accuracy and specificity, but these improvements do not automatically produce broader learning benefits. The value of customization appears to be more pronounced when learners act directly on explicit suggestions, whereas Socratic feedback may preserve a stronger role for learner-generated reasoning and monitoring. Rather than identifying one universally superior design, this study highlights the need to align agent configuration and feedback style with instructional goals while preserving learners’ active role in evaluating and using AI-generated feedback.

## Figures and Tables

**Table 1:** Overview of agent configuration under four groups.

Experimental Condition	Agent Type	Feedback Style	Key Configuration Differences
Condition 1	Customized	Socratic Feedback	Prompt + Knowledge Base (Textbook + Rubric) + Workflow; uses guiding questions strategy
Condition 2	General-purpose	Socratic Feedback	Relies solely on prompts; uses guiding questions strategy, with no external knowledge base
Condition 3	Customized	Directive Feedback	Prompt + Knowledge Base (Textbook + Rubric) + Workflow; uses direct identification of issues and suggestions strategy
Condition 4	General-purpose	Directive Feedback	Relies solely on prompts; uses direct identification of issues and suggestions strategy, no external knowledge base

**Table 2:** Comparison of the Two Instructional Design Tasks.

Task Element	Task 1: Week 1	Task 2: Week 2
Task theme	University classroom: Artificial Intelligence and Educational Applications	Middle school classroom: VR-supported project-based learning
Teaching context	Acting as a university instructor teaching the course Artificial Intelligence and Educational Applications to sophomore undergraduates majoring in educational technology	Acting as a middle school geography teacher teaching Grade 8 students on the topic of “Environmental Protection and Sustainable Development of Future Cities”
Lesson duration	90 min	80 min
Core task	Design a complete lesson plan to help students understand typical applications of AI in education, including GenAI-assisted learning, intelligent feedback, learning analytics, and prediction	Design a complete project-based lesson supported by VR technology, in which students use immersive VR to identify ecological problems and work in groups to propose and present improvement plans
Learner characteristics	Undergraduate students with basic knowledge of education and information technology, but limited prior learning about AI applications in education	Middle school students with interest in new technologies and group collaboration, as well as basic geography knowledge
Task difficulty	Equivalent: both tasks required students to present core instructional design elements, including learning objectives, activity design, and assessment methods	Equivalent: both tasks required students to present core instructional design elements, including learning objectives, activity design, and assessment methods

**Table 3:** Descriptive statistics for the four groups.

Dependent Variables	Subscales	Condition 1: Customized + Socratic	Condition 2: General-Purpose + Socratic	Condition 3: Customized + Directive	Condition 4: General-Purpose + Directive
Feedback Quality	Criteria-based relevance	4.02 (0.32)	3.96 (0.53)	4.20 (0.68)	4.02 (0.67)
	Specificity	3.87 (0.63)	3.50 (0.49)	4.24 (0.79)	3.73 (0.53)
	Accuracy	4.63 (0.45)	4.08 (0.65)	4.54 (0.54)	3.90 (0.51)
	Prioritization of essential features	3.65 (0.81)	3.38 (0.58)	3.96 (0.69)	3.67 (0.64)
	Supportive tone	4.04 (0.24)	4.04 (0.14)	4.00 (0.22)	4.00 (0.15)
Self-Regulatory Behaviors	Task orientation	4.00 (0.46)	4.00 (0.57)	3.57 (0.47)	3.75 (0.59)
	Comprehension monitoring	2.89 (1.30)	3.38 (1.25)	1.98 (1.06)	2.29 (1.18)
	Feedback regulation	2.46 (1.49)	3.00 (1.27)	2.89 (1.43)	2.48 (1.43)
Learning Experiences	Perceived Ease of Use	3.91 (0.66)	4.03 (0.52)	3.99 (0.77)	4.08 (0.39)
	Perceived Usefulness	4.01 (0.61)	4.05 (0.50)	3.95 (0.76)	4.06 (0.53)
	Intention to Use	4.03 (0.68)	4.02 (0.54)	4.00 (0.75)	4.02 (0.77)
	Cognitive Engagement	3.78 (0.59)	3.87 (0.53)	3.70 (0.57)	3.81 (0.50)
	Behavioral Engagement	4.10 (0.48)	4.13 (0.49)	4.16 (0.53)	4.23 (0.49)
	Emotional Engagement	3.73 (0.79)	3.94 (0.69)	3.84 (0.75)	3.99 (0.59)
	Critical Thinking Openness	3.90 (0.45)	3.99 (0.34)	3.96 (0.57)	4.04 (0.32)
	Reflective Skepticism	3.98 (0.56)	3.96 (0.37)	4.04 (0.49)	4.02 (0.45)
	Cognitive Load	2.88 (0.84)	2.85 (0.66)	3.01 (0.92)	3.24 (0.81)
Learning Outcomes	Gain scores	13.07 (8.37)	13.88 (6.28)	11.33 (6.05)	7.67 (3.45)

**Table 4:** Effects of agent type and feedback style on feedback quality.

	Dimension	Main Effect		Interaction
		Agent Type	Feedback Style	
**Feedback Quality**	Criteria-based relevance	*F*_(1,49)_ = 1.171, *p* = 0.284, *η*^2^*p* = 0.023	*F*_(1,49)_ = 1.204, *p* = 0.278, *η*^2^*p* = 0.024	*F*_(1,49)_ = 0.295, *p* = 0.589, *η*^2^*p* = 0.006
	Specificity	*F*_(1,49)_ = 10.492, *p* = 0.002 **, *η*^2^*p* = 0.176	*F*_(1,49)_ = 7.269, *p* = 0.010 **, *η*^2^*p* = 0.129	*F*_(1,49)_ = 0.403, *p* = 0.528, *η*^2^*p* = 0.008
	Accuracy	*F*_(1,49)_ = 28.561, *p* < 0.001 ***, *η*^2^*p* = 0.368	*F*_(1,49)_ = 1.848, *p* = 0.180, *η*^2^*p* = 0.036	*F*_(1,49)_ = 0.212, *p* = 0.647, *η*^2^*p* = 0.004
	Prioritization of essential features	*F*_(1,49)_ = 2.838, *p* = 0.098, *η*^2^*p* = 0.055	*F*_(1,49)_ = 10.635, *p* = 0.002 **, *η*^2^*p* = 0.178	*F*_(1,49)_ = 0.015, *p* = 0.901, *η*^2^*p* < 0.001
	Supportive tone	*F*_(1,49)_ = 0.003, *p* = 0.954, *η*^2^*p* < 0.001	*F*_(1,49)_ = 1.320, *p* = 0.256, *η*^2^*p* = 0.026	*F*_(1,49)_ = 0.005, *p* = 0.946, *η*^2^*p* < 0.001

*Note*: ** *p* < 0.01, *** *p* < 0.001.

**Table 5:** Effects of agent type and feedback style on self-regulatory behaviors.

	Dimension	Main Effect		Interaction
		Agent Type	Feedback Style	
**Self-regulatory behaviors**	Task orientation	*F*_(1,49)_ = 0.569, *p* = 0.454, *η*^2^*p* = 0.011	*F*_(1,49)_ = 14.328, *p* < 0.001 ***, *η*^2^*p* = 0.226	*F(*_1,49)_ = 0.971, *p* = 0.329, *η*^2^*p* = 0.019
	Comprehension monitoring	*F*_(1,49)_ = 2.150, *p* = 0.149, *η*^2^*p* = 0.042	*F*_(1,49)_ = 25.087, *p* < 0.001 ***, *η*^2^*p* = 0.339	*F*_(1,49)_ = 0.196, *p = 0*.660, *η*^2^*p* = 0.004
	Feedback regulation	*F*_(1,49)_ = 0.040, *p* = 0.843, *η*^2^*p* = 0.001	*F*_(1,49)_ = 0.042, *p* = 0.839, *η*^2^*p* = 0.001	*F*_(1,49)_ = 4.146, *p = 0*.047 *, *η*^2^*p* = 0.078

Note: * *p* < 0.05, *** *p* < 0.001.

**Table 6:** Effects of agent type and feedback style on learning experiences.

	Dimension	Main Effect		Interaction
		Agent Type	Feedback Style	
**Learning Experiences**	Perceived Ease of Use	*F*_(1,49)_ = 0.481, *p* = 0.491, *η*^2^*p* = 0.010	*F*_(1,49)_ = 0.896, *p* = 0.349, *η*^2^*p* = 0.018	*F*_(1,49)_ = 0.048, *p* = 0.828, *η*^2^*p* = 0.001
	Perceived Usefulness	*F*_(1,49)_ = 0.209, *p* = 0.649, *η*^2^*p* = 0.004	*F*_(1,49)_ = 0.154, *p* = 0.696, *η*^2^*p* = 0.003	*F*_(1,49)_ = 0.309, *p* = 0.581, *η*^2^*p* = 0.006
	Intention to Use	*F*_(1,49)_ = 0.001, *p* = 0.970, *η*^2^*p* = 0.000	*F*_(1,49)_ = 0.047, *p* = 0.830, *η*^2^*p* = 0.001	*F*_(1,49)_ = 0.047, *p* = 0.830, *η*^2^*p* = 0.001
	Cognitive Engagement	*F*_(1,49)_ = 0.534, *p* = 0.468, *η*^2^*p* = 0.011	*F*_(1,49)_ = 0.952, *p* = 0.334, *η*^2^*p* = 0.019	*F*_(1,49)_ = 0.026, *p* = 0.872, *η*^2^*p* = 0.001
	Behavioral Engagement	*F*_(1,49)_ = 0.145, *p* = 0.705, *η*^2^*p* = 0.003	*F*_(1,49)_ = 2.534, *p* = 0.118, *η*^2^*p = 0*.049	*F*_(1,49)_ = 0.063, *p* = 0.803, *η*^2^*p* = 0.001
	Emotional Engagement	*F*_(1,49)_ = 0.864, *p* = 0.357, *η*^2^*p* = 0.017	*F*_(1,49)_ = 1.631, *p* = 0.208, *η*^2^*p* = 0.032	*F*_(1,49)_ = 0.213, *p* = 0.646, *η*^2^*p* = 0.004
	Critical Thinking Openness	*F*_(1,49)_ = 0.624, *p* = 0.433, *η*^2^*p* = 0.013	*F*_(1,49)_ = 0.762, *p* = 0.387, *η*^2^*p* = 0.015	*F*_(1,49)_ = 0.002, *p* = 0.962, *η*^2^*p* = 0.000
	Reflective skepticism	*F*_(1,49)_ = 0.024, *p* = 0.877, *η*^2^*p* = 0.000	*F*_(1,49)_ = 1.669, *p* = 0.202, *η*^2^*p* = 0.033	*F*_(1,49)_ = 0.006, *p* = 0.940, *η*^2^*p* = 0.000
	Cognitive Load	*F*_(1,49)_ = 0.249, *p* = 0.620, *η*^2^*p* = 0.005	*F*_(1,49)_ = 6.222, *p* = 0.016 *, *η*^2^*p* = 0.113	*F*_(1,49)_ = 1.535, *p* = 0.221, *η*^2^*p* = 0.030

Note: * *p* < 0.05.

## Data Availability

The data presented in this study are openly available in the Mendeley Data database at https://data.mendeley.com/datasets/pjnv3mmh4c/1 (accessed on 6 April 2026).

## Supplementary Materials

The following supporting information can be downloaded at https://www.mdpi.com/article/10.3390/bs16071069/s1: Table S1: Instructional Design Evaluation Form; Table S2: Representative One-Turn Feedback Excerpts across the Four Experimental Conditions; Table S3: Coding Table-Feedback Quality; Table S4: Representative High and Low Scoring Excerpts for Feedback Quality; Table S5: Coding Table-Student’s Self-Regulatory Behaviors; Table S6: Representative High and Low Scoring Excerpts for Self-Regulatory Behaviors; Table S7: AI Agent Interaction Experience Questionnaire.

## Appendix A

**Table A1:** Thematic Structure with Category and Node Frequencies by Condition.

Theme	Categories (Frequency Count)	Nodes (Frequency Count)	C1	C2	C3	C4
Theme 1	Metacognitive Reflection (213) Dialogic Scaffolding (34) Cognitive Load Perception (42)	Deep Logic Reflection (157)	28	35	45	49
		Structural Issue Identification (56)	16	18	9	13
		Follow-up Prompting (34)	17	17	0	0
		Overload & Resistance (42)	9	9	14	10
Theme 2	Feedback Uptake & Translation (503) Critical Rejection Strategy (114) Agency Reassertion (85)	Substantive Revision (446)	90	77	122	157
		Selective Integration (57)	16	14	14	13
		Logic-based Rejection (114)	28	37	32	17
		Self-as-Decision-Maker (65)	21	21	12	11
		Interaction Strategies (20)	10	1	7	2
Theme 3	Pedagogical Efficacy (104) Contextual Anchoring (68) AI Role Perception (115) Scrutiny & Continuance (28)	Clear & Actionable Feedback (92)	31	27	18	16
		Professional Pedagogy (12)	9	2	1	0
		Contextual Deficit (68)	15	12	19	22
		AI as Co-Thinker (93)	27	30	16	20
		AI as Tool (22)	5	3	9	5
		Limitation Scrutiny (14)	2	6	2	4
		Continuance Intention (14)	7	5	2	0
