# Supplementary material for: The Effects of Agent Type and Feedback Style on Self-Directed Learning: A Mixed-Methods Study
Source: Behav Sci (Basel). 2026 Jun 30;16(7):1069. doi: 10.3390/bs16071069 (PMC13404235; doi:10.3390/bs16071069)
Supplement: Supplementary file 1 [file behavsci-16-01069-s001.zip › Supplementary Table S1.pdf]

Supplementary Table S1. Instructional Design Evaluation Form

Evaluation Dimension	Evaluation Criteria
**Completeness (20%)**	① Does the design include all the core components of the instructional design process: - Front-end analysis → Instructional objectives → Instructional activities → Instructional resources/environment → Assessment and feedback → Summary and improvement?<br>② Are the components logically connected and form a coherent chain, rather than being independent?
**Systematic Approach (20%)**	① Are the elements of instructional objectives, activities, resources, and assessment consistent and aligned, reflecting an overall design vision, rather than being pieced together arbitrarily?<br>② Does the design reflect a systematic arrangement, progressing from simple to complex, and from easy to difficult?
**Objectives and Learner Analysis (15%)**	**Objectives**: ① Are the objectives written with "action verb + condition + standard"? ② Are the objectives measurable and observable?<br>**Learner Analysis**: ① Does it consider students' age, interests, cognitive levels, etc.? ② Does it address learning differences (e.g., learning styles, language proficiency, etc.)?
**Teaching Strategies and Activities (15%)**	**Teaching Methods**: ① Are diverse strategies used (e.g., lecturing, group cooperation, inquiry-based learning, project-based learning, flipped classrooms, etc.)? ② Do the methods align with the learning objectives?<br>**Learning Activities**: ① Do the activities involve hands-on work, thinking, communication, and presentation? ② Are the activities authentic, challenging, and related to life or profession? ③ Is the teaching process complete (Introduction → Instruction → Practice → Summary)? Are the transitions between stages natural?
**Assessment and Feedback (10%)**	**Formative Assessment**: ① Are in-class assessments, instant feedback, peer assessments, etc., incorporated? ② Is the feedback specific, timely, and actionable?<br>**Summative Assessment**: ① Are diverse assessment methods used (e.g., exams, projects, presentations, portfolios)? ② Is there a balance between process and outcome?
**Resources and Environment (10%)**	**Teaching Resources**: ① Are appropriate textbooks, cases, and media selected? ② Is information technology/AI tools used reasonably (e.g., online assessments, personalized recommendations)?<br>**Learning Environment**: ① Is the classroom layout conducive to interaction (e.g., group work, discussion areas)? ② Is the necessary learning environment for introducing technology considered?
