# Supplementary material for: The Effects of Agent Type and Feedback Style on Self-Directed Learning: A Mixed-Methods Study
Source: Behav Sci (Basel). 2026 Jun 30;16(7):1069. doi: 10.3390/bs16071069 (PMC13404235; doi:10.3390/bs16071069)
Supplement: Supplementary file 1 [file behavsci-16-01069-s001.zip › Supplementary Table S2.pdf]

Supplementary Table S2. Representative One-Turn Feedback Excerpts across the Four Experimental Conditions

Note. The excerpts were selected from representative one-turn human–AI interactions in each experimental condition. All excerpts were anonymized and translated from Chinese into English. Minor language edits were made only to improve readability without changing the substantive meaning of the feedback.

C1 Customized + Socratic

Student:
I think these suggestions are feasible, but they seem to be somewhat difficult to implement.

Agent:
Indeed, implementing such a teaching strategy may involve some challenges. Let us discuss these difficulties step by step and try to find possible solutions.

1. **Clarifying team goals**: Ensuring that every student understands the team goals may require extra time for communication and explanation. You may consider using group discussion at the beginning of the lesson to allow students to participate jointly in setting goals, so that they are more likely to be engaged with and understand these goals.
2. **Encouraging autonomous contribution**: Encouraging students to contribute autonomously may require creating a safe and supportive environment where students feel that their opinions are respected. You can encourage students to speak by establishing a classroom culture that emphasizes respect and listening to everyone's views.
3. **Regular feedback**: Providing regular feedback may require you to invest more time and effort. You may consider using some tools or methods to help you collect and provide feedback more effectively, such as using online survey tools or giving brief oral feedback after group activities.
4. **Diversified activities**: Designing diversified activities may require more creativity and resources. You can try grouping students and allowing them to design activities themselves, or use existing teaching resources, such as project-based learning, to provide diverse learning experiences.

Do you think these methods help address the implementation difficulties? Or do you have any other ideas and strategies for responding to these challenges? We can discuss them together and find the solution that best fits your students.**C2 General-purpose + Socratic**

Student:
What you said makes sense. Do you think that the structure of my instructional design is also not complete enough? Does it lack learner analysis, teaching content analysis, teaching objective analysis, teaching methods, and teaching strategies?

Agent:
You have raised a very good point. Indeed, the systematicity of instructional design involves multiple aspects, including learner analysis, teaching content analysis, teaching objective analysis, teaching methods, and teaching strategies. These elements together constitute a complete instructional design framework.

1. **Learner analysis**: You have already mentioned students' background knowledge, which is a good start. However, have you thought about their attitudes toward artificial intelligence, learning styles, or prior experiences? These factors may all influence their learning process.
2. **Teaching content analysis**: Your plan mentions several application scenarios of artificial intelligence in education, which is a good starting point. However, have you further analyzed the progression of these contents and their relevance to the teaching objectives?
3. **Teaching objective analysis**: Your plan implies that students should understand the applications of artificial intelligence in education, but are there more specific and measurable teaching objectives? For example, what should students be able to do by the end of the lesson?
4. **Teaching methods and teaching strategies**: You proposed task-driven learning and group presentations, both of which are good interactive learning strategies. However, have you considered how to choose the most appropriate teaching methods according to the learning content and student characteristics?

By analyzing these aspects in more detail, your instructional design will become more systematic and complete. Could you share your specific thoughts on these aspects?**C3 Customized + Directive**

Student:
I think this revision is reasonable, and I accept this revision suggestion.

Agent:
Very good. Accepting this revision suggestion is a step forward. Now, your instructional design already includes more specific and more assessable expected learning outcomes, which will help you implement and evaluate the teaching activities more effectively.

Next, we can continue discussing the next dimension in your instructional design: assessment and feedback.

In terms of assessment and feedback, your instructional design should include how to evaluate whether students have achieved the expected learning outcomes, as well as how to provide feedback to promote student learning. This includes formative assessment (assessment conducted during the teaching process) and summative assessment (assessment conducted after the teaching process).

Suggestions for assessment and feedback:

1. **Formative assessment**: After the VR experience session, you may ask students to submit the list of environmental problems they recorded, along with their preliminary solutions to these problems. This can serve as part of the formative assessment. Based on what they submit, you can provide immediate feedback to help them improve and deepen their solutions.
2. **Summative assessment**: At the end of the course, you can design a comprehensive product, such as a project report or a related report, to let students demonstrate their understanding of environmental problems and their solutions. As a summative assessment, you can evaluate whether they have achieved the teaching objectives based on their presentation.
3. **Feedback mechanism**: Make sure that you have a clear feedback mechanism so that students know how they performed and how they can improve. This may take the form of written feedback, one-on-one discussion, or group feedback.

If these contents have not yet been included in your instructional design, you can add them. For example, you can add the following content to the document:

"Assessment and Feedback:
• Formative assessment: After the VR experience session, students submit a list of environmental problems and preliminary solutions. Based on their submissions, I will provide written feedback, indicating whether their observations are accurate, whether their solutions are feasible, and offering suggestions for improvement.
• Summative assessment: At the end of the course, students will give an oral presentation to demonstrate their understanding of environmental problems and the solutions they proposed. I will score them according to the content of their presentation and their presentation skills, and provide feedback highlighting what they did well and the areas that need improvement."

If you have already included these contents, we can further discuss how to optimize your assessment and feedback strategy. If you have any questions or need further help, please feel free to let me know.**C4 General-purpose + Directive**

Student:
Hello, thank you very much for the suggestions you provided from the dimension of "objectives and learner analysis." I plan to make the following adjustments:
Depth of learner analysis: Based on students' existing practical experiences, such as "garbage classification" and "energy conservation and emission reduction," I will further analyze students' specific needs and interests. For example, they are curious about new technologies such as "smart transportation" and "green energy," but they lack a comprehensive perspective and transfer ability when systematically thinking about "sustainable development of future cities."
Specificity of teaching objectives: I will further specify the objectives. For example, I will revise "improving the ability to transfer from reality to the future" into "students will be able to identify the connections between environmental protection practices in real life and environmental protection measures in future cities."
Measurability of teaching objectives: I will identify measurable behaviors corresponding to

each objective. For example, “discussion and analysis ability” can be reflected through the frequency and depth of students’ participation in group discussions, and “proposing solutions” can be measured through the completeness and innovativeness of the presentation outcomes.

Agent:
Your adjustment plan responds well to the requirements of the “objectives and learner analysis” dimension. By further refining learner analysis and teaching objectives, you have ensured the specificity and measurability of the teaching objectives. This adjustment will help evaluate students’ learning outcomes more effectively in actual teaching.

Now, we can move to the fourth dimension: teaching strategies and activities. In this dimension, we need to ensure that the selected teaching strategies and activities can effectively support the achievement of the teaching objectives.

1. **Appropriateness of teaching strategies**: You mentioned the use of project-based learning and cooperative group learning. Please ensure that these strategies can meet students’ needs and promote their in-depth understanding and exploration of the theme of “environmental protection and sustainable development of future cities.”
2. **Targeted design of activities**: Your teaching process includes multiple activity segments, such as “project experience and discussion” and “group presentation and interaction.” Please ensure that each activity can specifically support the achievement of the teaching objectives. For example, VR experience can help students identify problems, and group discussion can promote analysis and proposal development.
3. **Feasibility of activity implementation**: Considering the current teaching environment and resource constraints, ensure that the designed activities are feasible in practice. For example, make sure that the VR devices and software can meet the teaching needs and that the teacher can effectively manage and guide group activities.
4. **Connection between activities**: You have already mentioned adjusting the connection between different segments, which is very important. Please ensure that the transitions between activities are natural, so that students can move smoothly from one activity to the next and maintain the continuity of learning.

Please make corresponding adjustments to your instructional design based on these suggestions to ensure the appropriateness, targetedness, and feasibility of the teaching strategies and activities. After completing this, we can continue to discuss the next dimension.
