# Supplementary material for: The Effects of Agent Type and Feedback Style on Self-Directed Learning: A Mixed-Methods Study
Source: Behav Sci (Basel). 2026 Jun 30;16(7):1069. doi: 10.3390/bs16071069 (PMC13404235; doi:10.3390/bs16071069)
Supplement: Supplementary file 1 [file behavsci-16-01069-s001.zip › Supplementary Table S3.pdf]

Supplementary Table S3. Coding Table-Feedback Quality

Rating Dimension	Dimension Description	Rating Description (1–5 Points)
**Criteria-based Relevance**	Whether the feedback clearly refers to core evaluation standards in instructional design (such as teaching objectives, learning activities, assessment methods, learner characteristics, etc.), rather than being generic.	**1 point:** All feedback is general, with no reference to any instructional design standards (e.g., "This can be changed," "The content is not good enough").<br>**2 points:** Most feedback is general, with one piece of feedback vaguely mentioning an instructional design standard (e.g., "The objective is a bit unclear," but without explaining why it's unclear).<br>**3 points:** Half of the feedback clearly references instructional design standards, with the other half being general (e.g., "The activity lacks time allocation" vs "This can be improved").<br>**4 points:** Most feedback clearly references instructional design standards, with a few being general (e.g., "The objective does not match the activity," "The assessment should be more specific").<br>**5 points:** All feedback clearly and consistently references core instructional design standards, with no general expressions (e.g., "This objective does not meet the measurability principle," "The activity design does not reflect cognitive progression").
**Specificity**	Whether the feedback provides clear, actionable suggestions for improvement that are directly linked to the student's specific design content (such as objectives, activities, assessments, etc.), rather than being vague.	**1 point:** No specific student work content is referenced, and no actionable suggestions are given aligned with instructional design standards or task requirements (e.g., "This can be changed," "The content is not good enough").<br>**2 points:** Very few references to specific student work, or lacking actionable next-step suggestions (e.g., "The objective needs to be more specific," but without explaining how).<br>**3 points:** Half of the feedback references specific design content (e.g., "Objective on page 2"), but the suggestions are vague or do not clearly specify an action path; the other half is general.<br>**4 points:** Most feedback clearly references specific design content (e.g., "Activity 1 lacks time allocation") and provides usable suggestions; a few suggestions are unclear or not fully linked.<br>**5 points:** Always references specific student outputs or design content (e.g., "In your objective, you mention 'increase interest,' but do not explain how to measure it") and provides clear, immediately actionable improvement suggestions (e.g., "Suggest changing it to 'Students should be able to state three influencing factors'"), with some even including examples or step-by-step instructions.
**Accuracy**	Whether the feedback accurately understands and evaluates the	**1 point:** All feedback is inaccurate and irrelevant to the student's

	student’s instructional design content.	assignment (e.g., commenting "The activity is too long" when it is actually a single-page design).<br>**2 points:** Most feedback is inaccurate, with only a few reflecting the actual situation (e.g., misreading the objective or misjudging the activity logic).<br>**3 points:** Some feedback is accurate, but one or more pieces of feedback are clearly inaccurate (e.g., "The objective and activity do not match," but they actually do).<br>**4 points:** Most feedback is accurate, with only one piece slightly inaccurate (e.g., "Suggested adding an assessment section" is reasonable, but the expression is not rigorous).<br>**5 points:** All feedback is accurate and correctly identifies and evaluates the student’s design content (e.g., "The objective 'increase interest' is not measurable" → which it indeed is not).
**Prioritization of Essential Features**	Whether the feedback prioritizes core issues in instructional design (e.g., alignment of objectives-activities-assessment, learner compatibility, etc.), and provides achievable, developmental suggestions for the student's current level, rather than making unrealistic demands.	**1 point:** Feedback is too difficult for the student's current level or completely ignores any core instructional design issues (e.g., only saying "Make the format neat").<br>**2 points:** Most feedback is unfeasible, non-essential, or inappropriate (e.g., suggesting adding a lot of new activities when the student has not yet mastered basic structure).<br>**3 points:** Half of the feedback focuses on core issues (e.g., misalignment of objectives and activities), while the other half addresses secondary or non-essential issues (e.g., suggesting adding more details); some suggestions are unachievable.<br>**4 points:** Most feedback focuses on one of the most appropriate points for improvement (e.g., "The objective does not match the activity"), with reasonable and actionable suggestions, and only a few points deviate.<br>**5 points:** All feedback focuses on the key issues that need to be solved first (e.g., "The objective is not measurable") and provides immediately actionable developmental suggestions (e.g., "Suggest changing it to 'Students should be able to state three influencing factors'").
**Supportive Tone**	Whether the AI feedback uses respectful, encouraging, and constructive language aimed at motivating the student to make revisions and enhancing their sense of self-efficacy, rather than being critical, negative, or anxiety-inducing.	**1 point:** Feedback is mainly critical, accusatory, or dismissive (e.g., "This is wrong," "Too bad"), with no positive reinforcement or encouragement, possibly damaging the student’s confidence.<br>**2 points:** Occasional neutral expressions, but the overall tone is negative or indifferent (e.g., "Needs to be rewritten," "Does not meet the requirements"), with no acknowledgment of the student's effort.<br>**3 points:** Some feedback includes praise or encouragement (e.g.,

		"The objective direction is right"), but there is still a lot of directive or judgmental language (e.g., "You should...").<br>**4 points:** Most feedback uses a constructive tone, acknowledging strengths before suggesting improvements (e.g., "Your activity design is clear, and it would be even better if the objective were more specific"), with a gentle tone.<br>**5 points:** Always uses respectful, encouraging, and supportive language, emphasizing growth potential (e.g., "You've identified learner needs well; next, you could try making the objective more measurable"), effectively promoting reflection and willingness to improve.
