# Supplementary material for: The Effects of Agent Type and Feedback Style on Self-Directed Learning: A Mixed-Methods Study
Source: Behav Sci (Basel). 2026 Jun 30;16(7):1069. doi: 10.3390/bs16071069 (PMC13404235; doi:10.3390/bs16071069)
Supplement: Supplementary file 1 [file behavsci-16-01069-s001.zip › Supplementary Table S4.pdf]

Supplementary Table S4. Representative High and Low Scoring Excerpts for Feedback Quality

Dimension	Score	Illustrative Excerpt	Brief Justification
Criteria- based relevance	High (5)	*Student:* "In my instructional design, the purpose of each session is not clearly stated. Do you have any suggestions?"<br>*AI:* "It is very important to clarify the purpose of each session in instructional design. Here are some suggestions:<br>1. Align with objectives: Directly link each session to the learning objectives.<br>2. Name each session: Give each session a clear, descriptive name.<br>3. State the purpose explicitly: At the start of each session, clearly explain its purpose and expected outcomes.<br>4. Design activities: Ensure activities are closely related to the session's purpose.<br>5. Logical connections: Ensure clear logical links between sessions, with each building on the previous.<br>6. Time allocation: Allocate time reasonably based on the purpose and importance of each session.<br>7. Feedback mechanisms: Include feedback mechanisms in session design.<br>8. Summary and reflection: At the end of each session, allow time for students to summarize and reflect.<br>9. Differentiated instruction: Provide multiple learning paths to accommodate different learning styles and abilities.	Every suggestion was grounded in established pedagogical standards (objective alignment, activity design, logical sequencing, time allocation, feedback mechanisms, differentiation, assessment, etc.). No generic or off- topic comments were present.

Dimension	Score	Illustrative Excerpt	Brief Justification
		10. Interaction and engagement: Increase student participation through group discussions, role- playing, etc.<br>11. Affective factors: Build emotional connections through storytelling, case studies, etc.<br>12. Demonstration and practice: For skill- based sessions, provide demonstrations and hands- on practice.<br>13. Assessment and testing: Include assessment and testing in session design to check achievement of learning objectives."	
	Low (3)	*Student:* "Here is my initial instructional design for a VR project- based learning lesson on 'Sustainable Development of Future Cities'."<br>*AI:* "I have read your design. Let us evaluate it dimension by dimension.<br>1. Completeness – You are missing learner analysis. Please add information about students' age, prior knowledge, learning styles, and how these affect your teaching strategies. (cites instructional design standard)<br>2. Systematicity – Your activities (VR exploration, brainstorming, presentation) lack a clear framework showing how they connect to each other and to the overall objectives. Please describe the logical links. (cites instructional design standard)<br>3. Teaching strategies and activities – Ensure that activities	Approximately half of the AI's suggestions referenced core instructional design standards (learner analysis, systematicity, assessment criteria). The other half either were generic ("ensure engagement", "participation of every student") or focused on overly detailed or secondary issues (classroom layout, AR/3D printing) that are not central to a draft instructional design. The mix of standard- based and non- standard comments aligns with the rubric descriptor for a score of 3.

Dimension	Score	Illustrative Excerpt	Brief Justification
		not only align with objectives but also fully engage students. Consider: participation of every student, differentiated instruction, immediate feedback, and meaningful technology integration. (partially standard- based, but generic "engagement" without concrete link to design principles)<br>4. Resources and environment – You need to describe the classroom layout, placement of VR equipment, technical troubleshooting procedures, etc. (overly detailed; not a core instructional design standard for a draft)<br>5. Assessment and feedback – Specify your assessment criteria, use multiple evaluation methods (self, peer, teacher), timeliness of feedback, and how you will adjust teaching based on feedback. (cites assessment standards)<br>6. Personalization and innovation – Provide different difficulty levels, foster creative thinking (e.g., 'creative workshop', 'design challenge'), and integrate other technologies (AR, 3D printing). (partially relevant but introduces non- essential elements at an early stage)"	
Specificity	High (5)	*Student*: "Can you create a complete instructional design for me?"<br>*AI*: "Of course. Here is a draft based on your information:<br>1. Course Objectives – Understand AI principles, develop critical thinking, enhance collaboration.<br>2. Course Content – Group tasks using AI tools, discussions	The AI produced a complete, structured design with seven enumerated sections, directly responding to the student's request. Each component was concrete and immediately usable.

Dimension	Score	Illustrative Excerpt	Brief Justification
		on AI advantages/challenges, after-class activities.<br>3. Teaching Methods – Group work with AI, representative sharing, online collaborative editing.<br>4. Assessment & Feedback – Written feedback, online discussion participation, group project reports.<br>5. Teaching Resources – Whiteboard, sticky notes, online docs.<br>6. Teaching Procedure – Introduce AI tools → group discussions → collect feedback → online editing → assign after-class activities → share experiences → teacher-led summary.<br>7. Summary & Reflection – Discuss long-term impact of AI in education.”	
	Low<br>(2)	*Student:* “Here is my instructional design for a middle school geography VR project- based learning lesson on ‘Sustainable Development of Future Cities’. Please give me some suggestions.”<br>*AI:* “I have read your design. Let us discuss it dimension by dimension.<br>1. Completeness – You need to add learner analysis. Please describe students‘ age, prior knowledge, learning styles, and how these affect your teaching strategies. (no specific guidance on *how* to write it)<br>2. Systematicity – You should clarify how each activity	The AI listed six broad areas for improvement but did not reference any specific content from the student’s design. Suggestions such as “add learner analysis”, “clarify logical links”, “describe classroom layout” were generic and provided no concrete examples, step- by- step guidance, or references to the student’s own text. No actionable revisions were offered. This matches the rubric descriptor for a score of 2.

Dimension	Score	Illustrative Excerpt	Brief Justification
		connects to the overall objectives and to each other. Please describe the logical links. (no concrete example or existing activity to revise)<br>3. Teaching strategies and activities – Ensure that activities fully engage students. Consider participation, differentiation, immediate feedback, and meaningful technology integration. (generic list, no actionable steps)<br>4. Resources and environment – Describe classroom layout, VR equipment placement, technical troubleshooting procedures. (overly detailed but still no specific reference to the student's current setup)<br>5. Assessment and feedback – Specify assessment criteria, use multiple evaluation methods, ensure timeliness, and show how you will adjust teaching based on feedback. (broad principles, no concrete rubric or example)<br>6. Personalization and innovation – Provide different difficulty levels, foster creative thinking, integrate other technologies (AR, 3D printing). (unrealistic for a draft, no link to the student's existing VR focus)"	
Accuracy	High (5)	*Student:* "Based on your suggestions, I designed interactive after-class activities and additional resources: reflective journals, group projects, online discussion platforms …"<br>*AI:* "Your design is very reasonable and thoughtful. By combining reflective journals, group projects, online	The AI correctly identified the key strengths ofthe student's design (combination of activities, promotion of collaboration and deep learning) and provided an accurate pedagogical judgment. No factual errors or misinterpretations were present.

Dimension	Score	Illustrative Excerpt	Brief Justification
		discussion platforms, and additional learning resources, you provide students with rich after-class opportunities and encourage interaction and collaboration. This design effectively promotes deep learning and critical thinking. ”	
	Low (3)	*Student:* "Here is my instructional design for a VR project- based learning lesson on 'Sustainable Development of Future Cities' for middle school geography."<br>*AI:* "I have read your design. Let us evaluate it dimension by dimension.<br>1. Completeness – You are missing learner analysis. Please add information about students' age, prior knowledge, learning styles, and how these affect your teaching strategies. (*Inaccurate*: The student's design already included a section 'Learner Analysis' with details about 8th graders' prior knowledge and learning styles – see student's earlier response.)<br>2. Teaching strategies and activities – Consider adding a VR device training session before the VR experience, as students may be unfamiliar with the technology. (*Partially accurate*: The student's design already contained 'Teacher explains VR equipment usage and precautions' in the procedure. The AI missed this.)<br>3. Resources and environment – Describe classroom layout, VR equipment placement, technical troubleshooting	The AI's suggestion to add learner analysis was inaccurate because the student's design already contained a detailed learner analysis section (as shown in the student's previous message). The suggestion to add a VR training session was partially inaccurate because the student had already included equipment instructions in the procedure. Other suggestions (e.g., adding classroom layout, assessment criteria) were accurate. The presence of at least one clearly inaccurate statement aligns with the rubric descriptor for a score of 3.

Dimension	Score	Illustrative Excerpt	Brief Justification
		procedures. (*Accurate*: These details were indeed missing.)<br>4. Assessment and feedback – Specify assessment criteria and use multiple evaluation methods. (*Accurate*: The student had only a brief description.)”	
Prioritization of essential features	High (5)	*Student:* “Here is my instructional design document. Could you review it and tell me what needs to be improved?”<br>*AI:* “I have read your instructional design document… Let us first focus on the first aspect: learning objectives. You have set knowledge objectives, skill objectives, and affective objectives… But I would like to ask: have you considered linking these objectives to students’ prior knowledge? For example, you mentioned that students already have basic knowledge of pedagogy and information technology. How can that prior knowledge help them achieve the objectives? Could there be a clearer way to express this?”<br>*Student:* “What changes do you suggest?”<br>*AI:* “In the objectives section, we can make some adjustments…<br>Knowledge objective: You could explicitly state how students will use their existing knowledge of pedagogy and information technology to understand the core concepts of AI…<br>Skill objective: You could emphasize how students will apply their existing IT knowledge to analyze advantages and	The AI immediately identified instructional objectives as the most critical issue, prioritized it over minor concerns, and provided concrete, actionable revisions for each objective. No distracting or low- priority suggestions were offered.

Dimension	Score	Illustrative Excerpt	Brief Justification
		discuss challenges …<br>Affective objective: You could emphasize how students will use their prior knowledge to develop an objective and rational attitude toward AI in education…”	
	Low (2)	*Student*: “Here is my initial instructional design for a VR project- based learning lesson on ‘Sustainable Development of Future Cities’.”<br>*AI*: “I have read your design. Let us evaluate it dimension by dimension.<br>1. Completeness – You are missing learner analysis. Please add information about students‘ age, prior knowledge, learning styles, and how these affect your teaching strategies.<br>2. Systematicity – Your activities (VR exploration, brainstorming, presentation) lack a clear framework showing how they connect to each other and to the overall objectives. Please describe the logical links.<br>3. Teaching strategies and activities – Ensure that activities not only align with objectives but also fully engage students. Consider: participation of every student, differentiated instruction, immediate feedback, and meaningful technology integration.<br>4. Resources and environment – You need to describe	The AI listed six broad areas (completeness, systematicity, teaching strategies, resources, assessment, personalization) without prioritizing. The student had only submitted an initial draft, yet the AI demanded extensive additions (classroom layout, technical troubleshooting, AR/3D printing, creative workshops) that are secondary to core instructional design elements. Feedback was overwhelming and not focused on the most critical immediate issue (e.g., learner analysis or objective alignment).

Dimension	Score	Illustrative Excerpt	Brief Justification
		the classroom layout, placement of VR equipment, technical troubleshooting procedures, etc.<br>5. Assessment and feedback – Specify your assessment criteria, use multiple evaluation methods (self, peer, teacher), timeliness of feedback, and how you will adjust teaching based on feedback.<br>6. Personalization and innovation – Provide different difficulty levels, foster creative thinking (e.g., 'creative workshop', 'design challenge'), and integrate other technologies (AR, 3D printing)."	
Supportive Tone	/	All students received ratings of 4-4.5 on this dimension. A representative example at the 4- level ("Your design is very reasonable and thoughtful…"), which demonstrates consistently encouraging and constructive language.	/

Note. The excerpts presented above are illustrative only. All scores were assigned holistically based on each student's complete interaction log, which typically contained 10-30 exchanges, rather than on the isolated excerpts shown here. These excerpts are intended to exemplify the type of feedback quality characteristic of each performance level, not to serve as the sole basis for scoring.
