# Supplementary material for: The Effects of Agent Type and Feedback Style on Self-Directed Learning: A Mixed-Methods Study
Source: Behav Sci (Basel). 2026 Jun 30;16(7):1069. doi: 10.3390/bs16071069 (PMC13404235; doi:10.3390/bs16071069)
Supplement: Supplementary file 1 [file behavsci-16-01069-s001.zip › Supplementary Table S5.pdf]

Supplementary Table S5. Coding Table-Student's Self-Regulatory Behaviors

Rating Dimension	Dimension Description	Rating Description (1–5 Points)
**Task Orientation**	Whether the student's interactions are centered around the instructional design task goals, and whether the questions contribute to task progress.	**1 point:** Interactions clearly deviate from the instructional design task, with scattered or irrelevant questions (e.g., asking AI "What is your favorite color?").<br>**2 points:** Some relation to the task, but the goal is unclear or the conversation frequently strays off topic (e.g., starting with objectives, then shifting to discussing AI capabilities).<br>**3 points:** Generally centered around the task, but there is some distraction or failure to stay focused on the core issues (e.g., asking about objectives, format, and AI capabilities simultaneously).<br>**4 points:** Interactions clearly focus on the instructional design task, with questions being specific and coherent (e.g., progressively asking about the alignment between objectives, activities, and assessments).<br>**5 points:** Interactions consistently adhere to the instructional design goal, with questions progressing logically, reflecting strong goal orientation and task advancement awareness (e.g., "I want to clarify the objectives first, then look at the activities" or "The objective is clear now, so next I want to focus on the assessment").
**Comprehension Monitoring**	Whether the student actively monitors their understanding of the AI feedback through questioning, clarification, or paraphrasing.	**1 point:** No clarification or follow-up questioning, only passively receiving feedback.<br>**2 points:** Only simple confirmatory questions (e.g., "Is this correct?" "Is that okay?"), without in-depth inquiry.<br>**3 points:** Some clarification or follow-up questions, but mostly surface-level understanding (e.g., "What do you mean by 'the objective needs to be more specific'?").<br>**4 points:** Actively clarifying or deeply probing key feedback content (asking only "why/how" questions, e.g., "Why is this objective unmeasurable?" "Can you give an example?").<br>**5 points:** Continuously and repeatedly deepening their understanding of the design issues through questioning and clarification, demonstrating active monitoring and in-depth exploration (e.g., "So, you mean the issue with my objective is that it's not measurable, right?" or "Is this revision in line with the standard you just mentioned?").

**Feedback Regulation**	Whether the student reflects on, revises, and adjusts their subsequent interaction strategies based on AI feedback.	**1 point:** Mechanically accepting or completely ignoring feedback, with no regulatory behavior.<br>**2 points:** Expressing vague modification intentions (e.g., "I'll change it"), but without specific actions.<br>**3 points:** Explaining how they will revise (e.g., "I'll make the objective more specific"), but without showing strategy adjustment.<br>**4 points:** Demonstrating a "feedback → revision → revalidation" loop in the interaction (e.g., "I revised the objective as you suggested, what do you think now?").<br>**5 points:** Actively comparing different options, explaining the reasoning behind their choices, and dynamically adjusting interaction strategies based on feedback (e.g., "I tried two versions of the objective, this one is more measurable, so I chose it").
