# Supplementary material for: The Effects of Agent Type and Feedback Style on Self-Directed Learning: A Mixed-Methods Study
Source: Behav Sci (Basel). 2026 Jun 30;16(7):1069. doi: 10.3390/bs16071069 (PMC13404235; doi:10.3390/bs16071069)
Supplement: Supplementary file 1 [file behavsci-16-01069-s001.zip › Supplementary Table S6.pdf]

Supplementary Table S6. Representative High and Low Scoring Excerpts for Self-Regulatory Behaviors

Dimension	Score	Illustrative Excerpt	Brief Justification
Task orientation	High (5)	"I plan to ask questions during case instruction and guide group discussions … to achieve knowledge and skill objectives; and when discussing AI advantages and challenges … to achieve affective objectives."	Interaction consistently focused on goals, with stepwise progression and strong task advancement. The student explicitly linked discussion strategies to different learning objectives.
	Low (3)	After the AI provided multiple suggestions for improving the instructional design (e.g., deepening discussion and integrating perspectives), the learner did not ask any clarification questions or attempt to verify understanding, responding only with brief acknowledgments.	The learner does not engage in clarification or verification of the AI's feedback. There is no evidence of active monitoring of understanding, indicating passive reception.
Comprehension monitoring	High (4)	"I have not considered this aspect yet. Do you have any suggestions? … and how should I evaluate students' presentation quality and their performance during discussions?"	Active clarification and follow-up on key feedback. The student acknowledged a gap, requested suggestions, and posed a specific evaluation question, but without iterative cycles typical of score 5.
	Low (1)	"Just give me a revised example directly."	No clarification or follow-up; passively received feedback. The student asked for a direct solution with no self-generated inquiry.
Feedback regulation	High (5)	"I think this approach fits my class … your evaluation plan could be more specific, rather than a single statement; it would be better to refine it into checklist-style options."	Active comparison of options, explanation of choices, and dynamic strategy adjustment. The student critically evaluated the suggestion and proposed a more specific format.
	Low (1.5)	"Learner analysis … please expand this section of learner analysis."	Mechanical repetition of AI's instruction with no reflection, revision planning, or strategic adjustment. The behavior fell between scores 1 and 2.

Note. The excerpts presented above are illustrative only. All scores were assigned holistically based on each student's complete interaction log, which typically contained 10-30 exchanges, rather than on the isolated excerpts shown here. These excerpts are intended to exemplify the type of self-regulatory behaviors characteristic of each performance level, not to serve as the sole basis for scoring.
