# Supplementary material for: The Effects of Agent Type and Feedback Style on Self-Directed Learning: A Mixed-Methods Study
Source: Behav Sci (Basel). 2026 Jun 30;16(7):1069. doi: 10.3390/bs16071069 (PMC13404235; doi:10.3390/bs16071069)
Supplement: Supplementary file 1 [file behavsci-16-01069-s001.zip › Supplementary Table S7.pdf]

Supplementary Table S7. AI Agent Interaction Experience Questionnaire

1. Perceived Ease of Use (a1–a4)

a1. I can easily use the generative AI tool to get feedback on my assignments.

a2. The process of interacting with it is clear, logical, and easy to understand.

a3. I encounter very little difficulty while using it.

a4. Overall, it is easy to get started with it in this task.

2. Perceived Usefulness (b1–b8)

b1. I enjoy the process of receiving assignment feedback through this AI.

b2. The AI feedback motivates me to improve my instructional design further.

b3. With the AI feedback, I feel more confident in my instructional design abilities.

b4. The AI feedback helped me identify issues or shortcomings in my assignment.

b5. It noticeably improved the quality of my instructional design.

b6. It helped me complete revisions to my instructional design more quickly.

b7. It made my revisions to the instructional design more precise.

b8. Overall, it was useful for completing this task.

3. Willingness to Use (c1–c4)

c1. I believe it is suitable as a tool for assisting with learning and improving assignments.

c2. I would like to use this tool to get feedback on assignments in other courses.

c3. I would use this kind of AI agent as much as possible in my learning and work, given the opportunity.

c4. I plan to continue using it in subsequent tasks.

4. Cognitive Engagement (d1–d5)

d1. I relate the AI suggestions to the instructional design theories I have learned.

d2. Before making changes, I make a list of the revisions to be made.

d3. When encountering problems, I try different revision approaches.

d4. I explain “why this change is necessary” in my own words, rather than simply copying the suggestions.

d5. I compare the feasibility of different revision plans before deciding which one to adopt.

5. Behavioral Engagement (e1–e5)

e1. While interacting with the AI, I can stay focused and keep making progress.

e2. Even if the AI is inaccurate at first, I will rephrase my questions or add more information to continue the conversation.

e3. I actively ask the AI for specific suggestions or examples to help me make revisions.

e4. I implement the AI's suggestions in the document and carefully record the changes.

e5. I can complete this round of revisions and submit them within the given time.

6. Emotional Engagement (f1–f5)

f1. I am interested in using AI to revise my instructional design.

f2. This process usually makes me feel happy.

f3. I often feel bored during this process. (reverse item)

f4. I gain something from this process.

f5. I am generally satisfied with the final product after revisions.

7. Critical Open-mindedness (g1–g7)

g1. During the interaction, I try to think about how to revise the instructional design from a holistic perspective.

g2. I use the new ideas provided by the AI to adjust my instructional design.

g3. I consult multiple sources of information, not just rely on the AI feedback.

g4. When modifying the instructional design, I pay attention to different viewpoints or revision plans suggested by the AI.

g5. I seriously consider the AI's suggestions, even if they differ from my original instructional design ideas.

g6. Understanding the AI's feedback and suggestions is important to me.

g7. It is important to me to explain to others why I accept or reject the AI's feedback.

8. Reflective Doubt (h1–h4)

h1. After modifying the instructional design based on the AI's feedback, I often reflect on whether this revision truly improved my design.

h2. Before adopting the AI's suggestions, I usually check the source or rationale behind them, rather than just following them blindly.

h3. Before accepting the AI’s proposed revisions, I consider their impact on the overall instructional design and student learning outcomes.
h4. I often reflect on the decisions I made when revising my instructional design, so I can do better next time.

9. Cognitive Load (I1–I4)

I1. The way the AI expresses its feedback confuses me.
I2. I feel that some of the information in the AI’s feedback is irrelevant to completing the task.
I3. Understanding the AI’s feedback takes extra time and effort.
I4. To understand the AI’s feedback, I have to divert my attention away from the main task.
